# Changes in the Proteome of Xylem Sap in *Brassica oleracea* in Response to *Fusarium oxysporum* Stress

**DOI:** 10.3389/fpls.2016.00031

**Published:** 2016-02-01

**Authors:** Zijing Pu, Yoko Ino, Yayoi Kimura, Asumi Tago, Motoki Shimizu, Satoshi Natsume, Yoshitaka Sano, Ryo Fujimoto, Kentaro Kaneko, Daniel J. Shea, Eigo Fukai, Shin-Ichi Fuji, Hisashi Hirano, Keiichi Okazaki

**Affiliations:** ^1^Graduate School of Science and Technology, Niigata UniversityNiigata, Japan; ^2^Advanced Medical Research Center, Yokohama City UniversityKanazawa, Japan; ^3^Iwate Biotechnology Research CenterKitakami, Japan; ^4^Graduate School of Agricultural Science, Kobe UniversityKobe, Japan; ^5^Faculty of Bioresource Sciences, Akita Prefectural UniversityAkita, Japan

**Keywords:** *Brassica oleracea*, *F. oxysporum* f. sp. *conglutinans*, xylem sap, proteomics

## Abstract

*Fusarium oxysporum* f.sp. *conlutinans* (Foc) is a serious root-invading and xylem-colonizing fungus that causes yellowing in *Brassica oleracea*. To comprehensively understand the interaction between *F. oxysporum* and *B. oleracea*, composition of the xylem sap proteome of the non-infected and Foc-infected plants was investigated in both resistant and susceptible cultivars using liquid chromatography-tandem mass spectrometry (LC-MS/MS) after in-solution digestion of xylem sap proteins. Whole genome sequencing of Foc was carried out and generated a predicted Foc protein database. The predicted Foc protein database was then combined with the public *B. oleracea* and *B. rapa* protein databases downloaded from Uniprot and used for protein identification. About 200 plant proteins were identified in the xylem sap of susceptible and resistant plants. Comparison between the non-infected and Foc-infected samples revealed that Foc infection causes changes to the protein composition in *B. oleracea* xylem sap where repressed proteins accounted for a greater proportion than those of induced in both the susceptible and resistant reactions. The analysis on the proteins with concentration change > = 2-fold indicated a large portion of up- and down-regulated proteins were those acting on carbohydrates. Proteins with leucine-rich repeats and legume lectin domains were mainly induced in both resistant and susceptible system, so was the case of thaumatins. Twenty-five Foc proteins were identified in the infected xylem sap and 10 of them were cysteine-containing secreted small proteins that are good candidates for virulence and/or avirulence effectors. The findings of differential response of protein contents in the xylem sap between the non-infected and Foc-infected samples as well as the Foc candidate effectors secreted in xylem provide valuable insights into *B. oleracea*-Foc interactions.

## Introduction

The xylem vessel is formed via the programmed death of xylem tracheary elements, followed by connection of the elements to long tubes. The important function of xylem is transporting water and minerals from the root to the aerial tissues of the plant (De Boer and Volkov, 2003). Furthermore, the observed macromolecules in the xylem sap, such as carbohydrates and proteins, are thought to have function in the response against biotic and abiotic stresses (Biles and Abeles, 1991; Satoh et al., 1992; Masuda et al., 1999; Sakuta and Satoh, 2000).

Xylem sap proteins come from two pathways; when xylem elements are dead, the protoplast is autolyzed and some of the proteins are released into the xylem. Since the differentiated xylem cells are not able to produce proteins by themselves, after completion of the xylem vessel, most xylem sap proteins are secreted from the stele cell in the root (Ligat et al., 2011). Earlier studies reported relatively abundant xylem sap proteins such as chitinase, peroxidase, and β-1,3-glucanases in cucumber (Masuda et al., 1999; Sakuta and Satoh, 2000) and tomato (Rep et al., 2002), using one-dimensional gel electrophoresis (1-DE). Subsequently, hundreds of proteins were detected in xylem sap in different species using 2-DE and high-throughput proteomics techniques (Alvarez et al., 2006; Djordjevic et al., 2007; Aki et al., 2008; Floerl et al., 2008; Dafoe and Constabel, 2009; Fernandez-Garcia et al., 2011; Ligat et al., 2011; Zhang et al., 2015). Ligat et al. (2011), in their study using liquid chromatography-tandem mass spectrometry (LC-MS/MS) and *Brassica* EST and cDNA sequences, classified 189 *B. oleracea* xylem sap proteins into eight of the nine functional classes previously defined for *Arabidopsis thaliana* cell wall proteins; most of them belong to those acting on carbohydrates (e.g., β-1,3-glucanases and chitinase), oxido-reducatses and proteases (29.2, 23.8, and 17.1%, respectively), which are also abundant/dominating protein classes in other plant xylem saps. Other small protein classes reported in Ligat et al. (2011), such as proteins related to lipid metabolism (4.9%), proteins with domains interacting with carbohydrates or proteins (e.g., lectin and protease inhibitor) (4.9%), miscellaneous proteins (8.5%) and proteins involved in signaling (5.5%), have also been described in *Glycine max, B. napus*, and *Oryza sativa* (Kehr et al., 2005; Djordjevic et al., 2007; Aki et al., 2008).

Many of xylem sap proteins contribute to a plant defense reaction, and the common presence of defense related proteins in plant xylem indicates the important role of xylem sap proteins in response to biotic and abiotic stresses in plants. Several xylem sap proteomics studies have suggested infection of pathogens induces some of the plant pathogenesis-related (PR) proteins. In oilseed rape, infection of *Verticillium longisporum* induced the PR-4 (chitinase) and PR-2 (β-1,3-glucanase) proteins in xylem sap (Floerl et al., 2008). β-1,3-glucanases and some other PRs were also induced in soybean by *F. virguliforme* infection (Abeysekara and Bhattacharyya, 2014). *F. oxysporum* f. sp. *lycopersici* (Fol) colonization induces PR-5 protein in both resistant and susceptible tomato, and the accumulation of PR-1, PR-2 as well as PR-3 concomitantly appeared with disease symptoms in susceptible tomato plants (Rep et al., 2002). In contrast, XSP10 which has similarity with lipid-transfer proteins (PR-14), declined in xylem sap with the infection of Fol, indicating that modification of secretion of this protein may be induced by the pathogen (Rep et al., 2003; Krasikov et al., 2011).

In addition to those PR proteins regulated in infected plants, xylem sap analyses also detected fungal proteins secreted into plant xylem during infection, including so-called effectors. Certain fungal effectors are specifically recognized by plant resistant gene (*R* gene) products and activate effector-triggered immunity (ETI) in the plant. The effectors recognized by R gene products are called avirulence (*Avr*) effectors and this genetic interaction between *R* and *Avr* genes is described as “gene-for-gene” theory (Flor, 1942; Jones and Dangl, 2006). A representative group of such effectors from vascular-invasion fungus are the Six (secreted in xylem) effectors which are commonly small and cysteine-rich secreted proteins in *F. oxysporum* species. Houterman et al. (2007) carried out a mixed xylem sap proteome analysis of Fol-infected tomato by 2-DE combined with mass spectrometry. Due to the lack of genomic information of Fol at that time, only seven Fol proteins including four Six (Six1-Six4) proteins were identified. Surprisingly, three of the four Six proteins (Six1, Six3, and Six4) have been functionally confirmed as Avr effectors (Avr3, Avr 2, and Avr1, respectively) in further studies (Rep et al., 2004, 2005; Houterman et al., 2008, 2009). After the whole genome sequence of Fol, more Six genes have been disclosed (Ma et al., 2010). In total, 14 Six genes have been identified so far in Fol (Schmidt et al., 2013). Some Six genes are also present in other formae speciales, for example, *Six1, Six4, Six8*, and *Six9* have homologs in f. sp. *conglutinans, Six6* also present in f. sp. *melonis* and f. sp. *radicis-cucumerinum, Six7* was also detected in f. sp. *lili* (Lievens et al., 2009; Thatcher et al., 2012).

Fusarium-wilt in *B. oleracea*, caused by *F. oxysporum* f. sp. *conglutinans* (Foc), is a destructive disease that results in severe losses in both yield and quality during *B. oleracea* production. Four homologs (*Six1, Six4, Six8*, and *Six9*) of the 14 so far reported Six genes in Fol have been found in Foc (Thatcher et al., 2012). Only Foc-*Six4* was confirmed as a virulence factor and the function of the remaining three Foc-*Six* homologs remain unknown (Thatcher et al., 2012; Kashiwa et al., 2013). On the other hand, major resistance locus against Foc has been mapped on the C7 chromosome of *B. oleracea*, and the resistant gene (named *Foc-Bo1*) was successfully cloned by map-based cloning (Pu et al., 2012; Shimizu et al., 2015). To date, however, there is no comprehensive study regarding the interaction between Foc and *B. oleracea*. The Avr effector in Foc is still unknown. Given the effectiveness of analyzing the xylem sap proteome in the tomato-Fol study (Rep et al., 2002, 2004), we therefore carried out a xylem sap proteomics analysis among resistant and susceptible cabbage, that were non-infected or infected with Foc, using in-solution digestion method before LC-MS/MS analysis. Whole genome sequencing for Foc was also performed with the expectation of improved accuracy in protein identification. The main purposes of this study are (1) to obtain a more comprehensive overview of the response in *B. oleracea* against Foc infection and (2) to investigate candidate effectors in Foc that contribute to virulence and/or avirulence toward *B. oleracea*.

## Materials and methods

### Plant and pathogen materials

Commercial cabbage F1 cultivars, YCR-Rinen (Nippon Norin Seed Co., Japan) and Delicious (Watanabe Seed Co., Japan) resistant and susceptible to Fusarium-wilt, respectively, were used in this study. YCR-Rinen contains the fusarium-wilt resistant gene (*Foc-Bo1*), while Delicious does not (Shimizu et al., 2015). The Cong: 1-1 strain of *F. oxysporum* f. sp. *conglutinans* (Foc), obtained from cabbage, was provided by Dr. Kadota (National Agricultural Research Center for Tohoku Region, Japan), and was used to prepare inocula. Inoculation was carried out as described in our previous paper (Pu et al., 2012).

### Visualizing of the infection process

To determine the optimal time for xylem sap collection, the infection site of Foc in *B. oleracea* was visualized by 5-bromo-4-chloro-3-indoxyl-α-L-arabinofuranoside (X-Ara) staining as reported by Diener (2012). Infected roots collected at 1 day post-infection (dpi), 3, 7, and 12 dpi were washed well with aqueous solution containing 0.1% Triton X-100 and 20 mM EDTA, pH 8.0. Peat attached to roots was removed carefully by sharp tweezers. The cleaned roots were then incubated with X-Ara in 40-fold volume staining solution (0.02% X-Ara, 10 mM EDTA, 1 mM K_3_Fe(CN)_6_, 0.1% Triton X-100 and 0.1 M sodium phosphate, pH 7.2) at 28°C overnight and the resulting blue color was observed in the Foc-invaded roots.

### Xylem sap collection

Four-week old plants were transplanted into Foc-infected or non-infected soil. Xylem sap collection was carried out 12 dpi when the susceptible plants show disease symptoms. Xylem sap collection was carried out according to the method reported by Buhtz et al. (2004). Briefly, xylem sap samples were obtained after cutting stems approximately 3 cm above soil level. To avoid the contamination from the phloem, cut-surface was thoroughly washed by distilled water and the first droplets appearing on the cut surface were removed with blotting paper. The following droplets resulting from “root pressure” were collected with a hand-held pipette. It was difficult to quantify the amount of proteins in the sap using the Bradford method, possibly due to low concentration of xylem sap proteins, which is consistent with the result of Ligat et al. (2011). Since protein concentration in the xylem sap is thought to be quite low, about 15 individual plants were used for collection of each sample, and the collected sap was pooled, frozen in liquid nitrogen, and stored at −80°C for further analysis.

### Protein precipitation

1 ml pooled xylem sap was concentrated by vacuum freezing centrifugal drying until approximately 200 μl. Then 1 mM HCl was added to the sample to adjust the pH around 4–5. Acetone precipitation was then carried out by adding 1 ml Acetone, followed by store at −80°C for 1 h. The precipitated proteins were collected by centrifugation for 30 min at 8000 g at 4°C. The pellet was air-dried and dissolved in 8 M urea and 2 M thiourea buffer. Protein concentration was determined by Bradford method. One-dimensional SDS-PAGE electrophoresis was carried out to get a primary image of protein patterns in each sample. Gel-separated proteins were visualized by silver staining (Wako Pure Chemical Industries, Osaka, Japan).

### LC-MS/MS analyses

Proper volume of the dissolved protein (about 1 μg) sample was reduced with 10 mM dithiothreitol (DTT) at 60°C for 30 min and carbamidomethylated with 25 mM iodoacetamide at room temperature for 15 min. Samples were diluted 2-fold with 50 mM ammonium bicarbonate followed by digestion with lysyl endopeptidase (Lys-C; Wako, Osaka, Japan) at 37°C for 3 h. Samples were then further diluted 2-fold with 50 mM ammonium bicarbonate, and subsequently digested with trypsin (Promega, Madison, MA, USA) at 37°C for 16 h. The resulting peptides were desalted using C18 stage tips according to the method published by Rappsilber et al. (2003). Label-free protein relative quantitation of each experimental plot with shotgun LC-MS/MS analysis was performed using equal amounts of purified tyrosine-phosphorylated peptides. LC-MS/MS analysis was performed on a LTQ Orbitrap Velos hybrid mass spectrometer (Thermo Fisher Scientific, Bremen, Germany) using the Xcalibur version 2.0.7. UltiMate® 3000 LC system (Dionex, LC Packings, Sunnyvale, CA, USA) was used to provide the gradient for online reversed-phase nano-LC at a flow rate of 300 nL/min. A C18 PepMap™ column (LC Packings) and a nanoscale C18 PepMap™ capillary column (75 μm id × 15 cm) (LC Packings) were used as analytical columns. The mobile phases were A (2% acetonitrile, 98% water, 0.1% formic acid) and B (95% acetonitrile, 5% water, 0.1% formic acid). Peptides were separated using a 145 min gradient program consisting a gradient of 2–33% B over 120 min. The full-scan mass spectra were measured from *m/z* 350 to 1200 in the positive-ion electrospray ionization mode on an LTQ Orbitrap Velos mass spectrometer (Thermo Fisher Scientific) operated in the data-dependent mode. The general mass-spectrometric conditions were as follows: spray voltage, 1.8 kV; capillary temperature, 250°C; normalized collision energy, 35.0%; isolation width, 2 *m/z*; activation time, 10 ms; activation Q, 0.25; dynamic exclusion, 180 s; resolution, 60,000; data-dependent mode, TOP15 strategy.

In-gel digestion followed by MS analysis identified the protein for each selected band. Proteins of interest were excised from silver stained gel and de-staining. Gel pieces were soaked in trypsin solution and incubation at 37°C for 16 h to digest after dehydration with acetonitrile. The resulting peptides were desalted using C18 stage tips. LC-MS/MS analysis was performed on a QSTAR XL mass spectrometer (Applied Biosystems, Foster City, CA, USA). To identify the sequence of peptides, peak lists were created using Analyst QS software. The obtained MS and MS/MS data were used for database searches using MASCOT Version 2.4.1 (Matrix Science, London, UK). The search parameters were as follows: trypsin digestion with two missed cleavages permitted, variable modifications (oxidation of methionine, carbamidomethylation of cysteine and propionamidation of cysteine), peptide mass tolerance for MS data ± 0.5 Da, and fragment mass tolerance ± 0.5 Da.

### Database construction and protein identification

Whole genome sequencing of Cong: 1-1 strain was carried out using a hybrid sequencing strategy, utilizing Hiseq1000 (Illumina) and GS Junior (Roche) next generation sequencers. The CLC Genomics Workbench program was used for *de novo* assembly of 6425 contigs with an average size of 8458 bp. The protein sequences were predicted by Augustus (http://bioinf.uni-greifswald.de/augustus/; Stanke and Morgenstern, 2005), and generated a Cong:1-1 protein sequences database with 17,009 predicted proteins (manuscript in preparation). The protein sequences of *B. rapa* subsp. *pekinensis* (41,212 proteins) and *B. oleracea* (1429 proteins) were downloaded from Uniprot (http://www.uniprot.org/). The Foc and plant protein sequences were combined together to generate a database. Two predicted proteins of the candidate Foc resistant genes, *FocBo1* in *B. oleracea* (GeneBank access to AB981182.1. Shimizu et al., 2015) and *FocBr1* in *B. rapa* (Bra012688. Shimizu et al., 2014) were also added into the database. Totally, the generated database contains 59,647 protein sequences for database searching. Additionally, we screened for contaminants by using a combined database of the contaminants database (http://www.matrixscience.com/help/seq_db_setup_contaminants.html) and our constructed protein database. As a result, several human proteins like trypsin and keratins were detected and then eliminated from the data. Furthermore, the same plant proteins and Foc-proteins were detected in the database search with/without the contaminants database.

To identify the sequence of peptides, peak lists were created using Proteome Discoverer (version 1.3). The obtained MS and MS/MS data were used for database searches using MASCOT Version 2.4.1 (Matrix Science, London, UK) with the following parameters: enzyme, trypsin; peptide mass tolerance, ±5 ppm; fragment mass tolerance, ±0.5 Da; max missed cleavages, 2; variable modifications, carbamidomethyl (C) and oxidation (M). In addition to the criterion of an FDR of 1% used as the acceptance criteria for all protein identifications, only proteins which were positively identified throughout the LC-MS/MS analysis (repeated three times) with score>=95 (at least two times among the repeats), were reported in this study. The proteins satisfying those criteria were also applied to protein quantitation in the Progenesis LC-MS deta analysis program (version 4.1, Non-linear Dynamics, Newcastle, UK). The protein content levels within each cultivar, i.e., between Del-Con and Del- Inf as well as between Ri-Con and Ri-Inf were compared in the Progenesis LC-MS analysis where the MS/MS data (three times repeat per experimental pot) were used for quantitation and the obtained normalized abundances of each peptide were subjected to statistical analysis using one-way analysis of variance (ANOVA). In this study, the detected feature was assumed to be statistically significant when the *p*-value for a given peptide was < 0.05 and minimum fold change was > = 2.

### Bioinformatics

The identified plant proteins were annotated based on Uniprot website information and the remaining uncharacterized proteins were further annotated with the InterPro domain annotation by searching the corresponding gene name in *Brassica* Database (http://brassicadb.org/brad/index.php). The corresponding *A. thaliana* genes were obtained from the known orthologs listed in the Brassica Database, or determined by a BLAST search against the Uniprot database. SignalP (http://www.cbs.dtu.dk/services/SignalP/) and TargetP http://www.cbs.dtu.dk/services/TargetP/) were used for sub-cellular localization prediction. The identified Foc proteins were annotated by NCBI protein BLAST (http://blast.ncbi.nlm.nih.gov/Blast.cgi) in Non-redundant protein sequences (nr) database using blastp algorithm.

Our MS/MS data was deposited to the PRIDE Archive, PRIDE Accession PXD003378 (http://www.ebi.ac.uk/pride/archive/). This Whole Genome Shotgun project has been deposited at DDBJ/EMBL/GenBank under the accession LPZQ00000000. The version described in this paper is version LPZQ01000000.

## Results

### Determining the time-point for harvesting the samples

**Figure 1:**
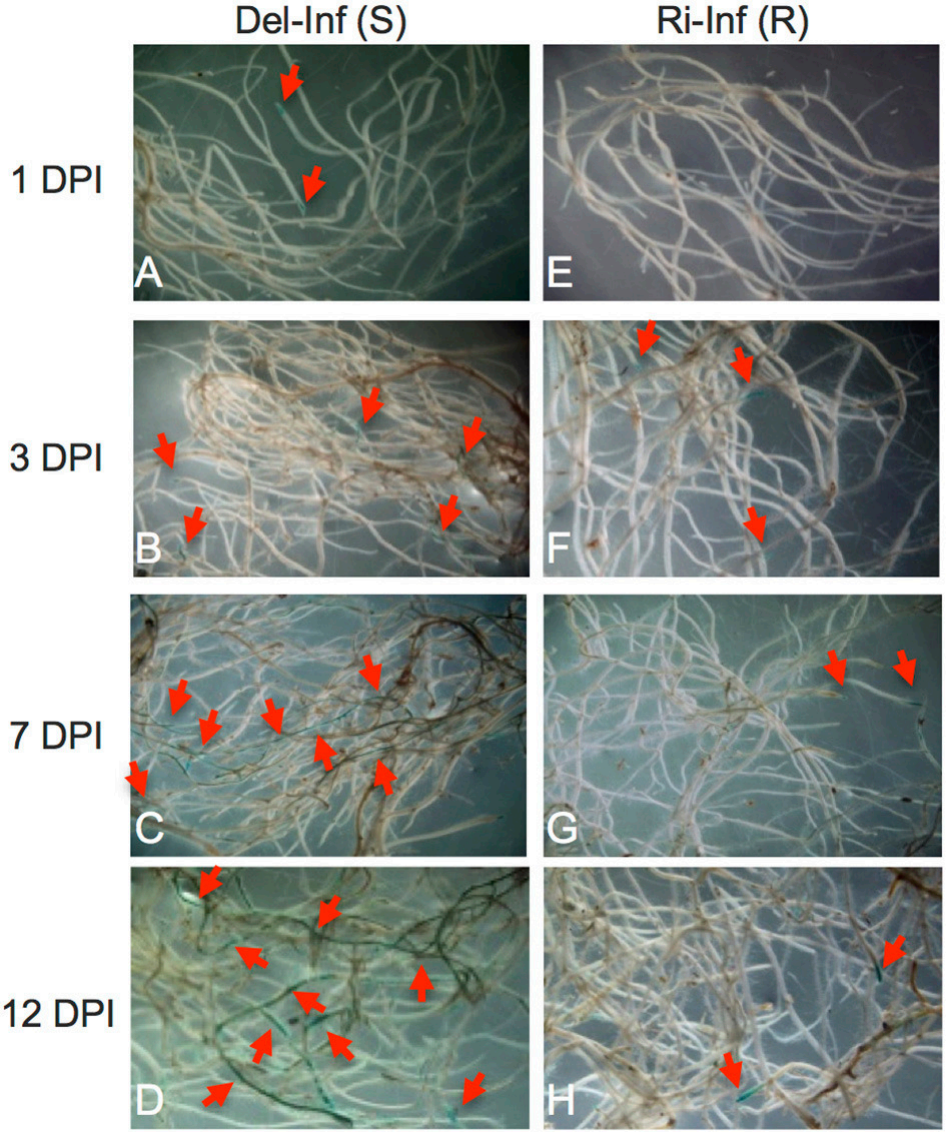
**5-Bromo-4-chloro-3-indoxyl-α-L-arabinofuranoside (X-Ara) staining of *Fusarium oxysporum* f. sp. *conglutinans* (Foc) infected root**. Roots of the Foc-infected YCR-Rinen (Ri-Inf, resistant) and Delicious (Del-Inf, susceptible) were collected 1, 3, 7, and 12 days post-infection (DPI) and stained overnight with X-Ara. Blue color designated by arrows indicates the Foc-infection sites. Panel **(A–D)** for the infected Delicious; Panel **(E–H)** for the infected YCR-Rinen.

Preliminary experiments indicated that YCR-Rinen was resistant, while Delicious showed susceptibility to Foc. 12-day-old plants were used for inoculation test and the symptoms were observed 6- to 7 dpi in our inoculation system (data not shown). The differences in the infected roots of YCR-Rinen and Delicious were visualized by X-Ara staining (Figure 1). In the susceptible cultivar (Delicious), subtle staining was observed at 1 dpi at the outer layers of the root apex and the lateral root primordial (LRP) (Figure 1A), supporting the previous study that the pathogen penetrates the surface of root apex and LRP, and traverses tissues outside the vascular cylinder in the primary phase of initial Foc penetration to *A. thaliana* (Diener, 2012). More infection points were observed at 3 dpi in the susceptible cultivar. The staining went through the root apex region, and developed into more basal tissue, especially in the vascular cylinder (Figure 1B). A thorough infiltration of the central vascular cylinder was observed at 7 dpi in the susceptible cultivar, and more extensive inroads into the road lines at 12 dpi was indicated by pervading of the precipitated blue (Figures 1C,D). The growth of Foc in host plant was associated with the development of yellowing symptoms in the aerial part: yellowing appeared from 6 to 7 dpi, developed as time went on, and finally the susceptible plant was dead.

The resistant cultivar showed no yellowing, but some plants showed stunted and retarded growth after infection. In the resistant cultivar, no infection was observed at 1 dpi (Figure 1E). Faint blue spots were detected from 3 dpi and the staining mostly confined in the root apex and LRP throughout the infection period tested in our study, indicating the restricted development of Foc in the resistant cultivar (Figures 1F–H). Early stage of infection showed limited growth of Foc, leading to less fungal proteins transported into the xylem of plants, while late stages of infection hinder xylem sap collection in susceptible plants, due to the advanced progression of symptoms. We therefore decided upon 12 days after inoculation as the appropriate point in time for the collection of xylem sap.

### SDS-PAGE and xylem protein identification via in-gel digestion

**Figure 2:**
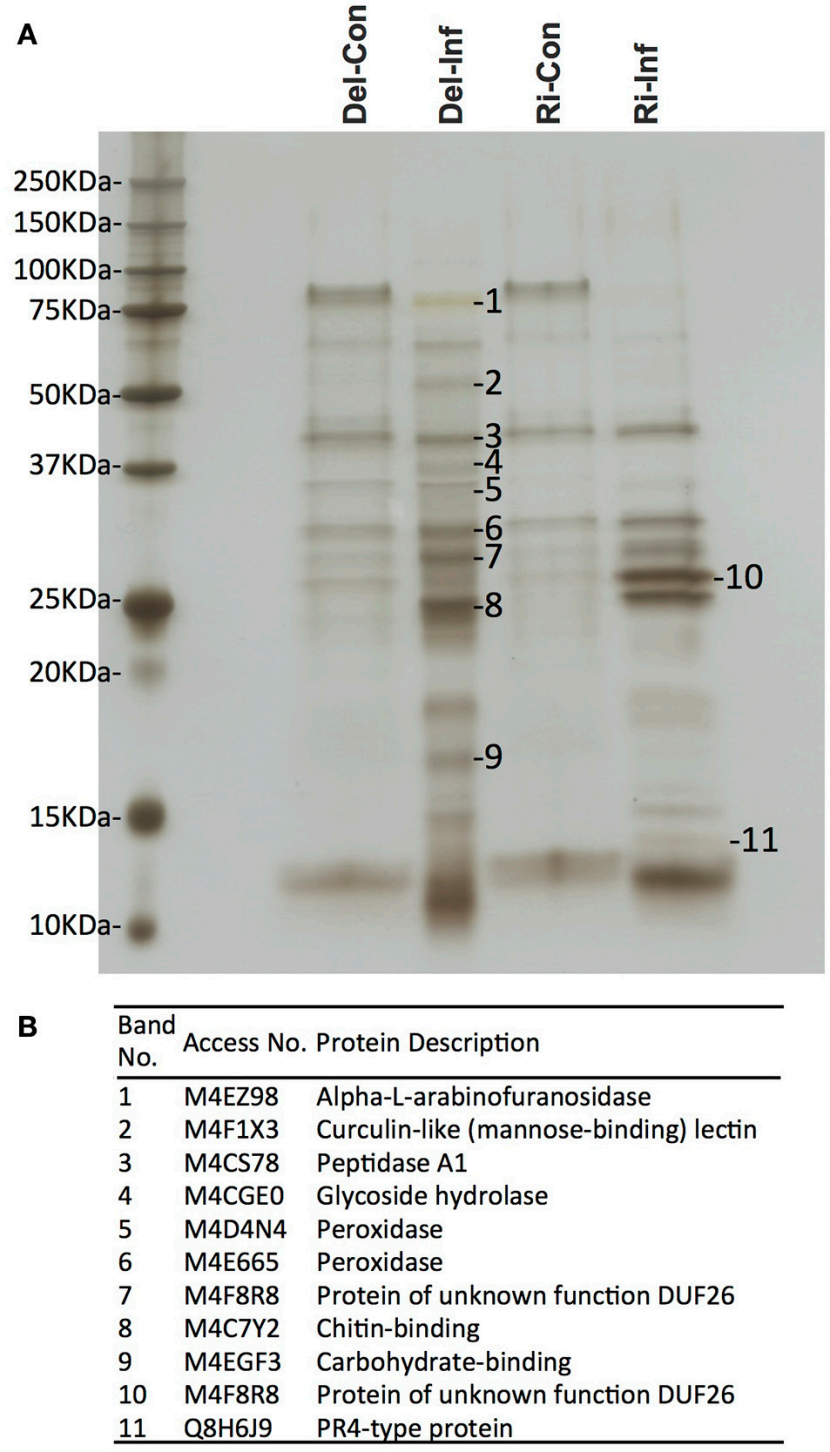
**Xylem sap proteins separated by one-dimensional SDS-PAGE followed by MS analysis**. Del-Con, Del-Inf, Ri-Con, and Ri-Inf indicate non-infected or Foc-infected plants of susceptible Delicious and resistant YCR-Rinen, respectively. **(A)** Several bands that show induced or repressed were selected for further analysis. **(B)** In-gel digestion followed by MS analysis identified the protein for each selected band.

The collected xylem was first separated on a one-dimensional SDS gel (Figure 2A). As expected, similar band patterns were observed in the non-infected samples in the different cultivars, YCR-Rinen and Delicious. On the other hand, the SDS gel analysis clearly showed that Foc infection induced different types of bands in the resistant and susceptible cultivars. We selected several major bands from the infected sample and proceeded with in-gel digestion so that only plant proteins such as arabinofuranosidase, glycoside hydrolase, and peroxidase etc. were detected with low score values (Figure 2B). Although the identification via the in-gel digestion analysis should be preliminary, this result indicates the overwhelming concentration of plant proteins in the xylem. The proteins identified by the in-gel digestion followed by LC/MS/MS were also identified by the following in-solution digestion analysis.

### Plant proteins identified in the non-infected and infected *B. oleracea* xylem sap

**Figure 3:**
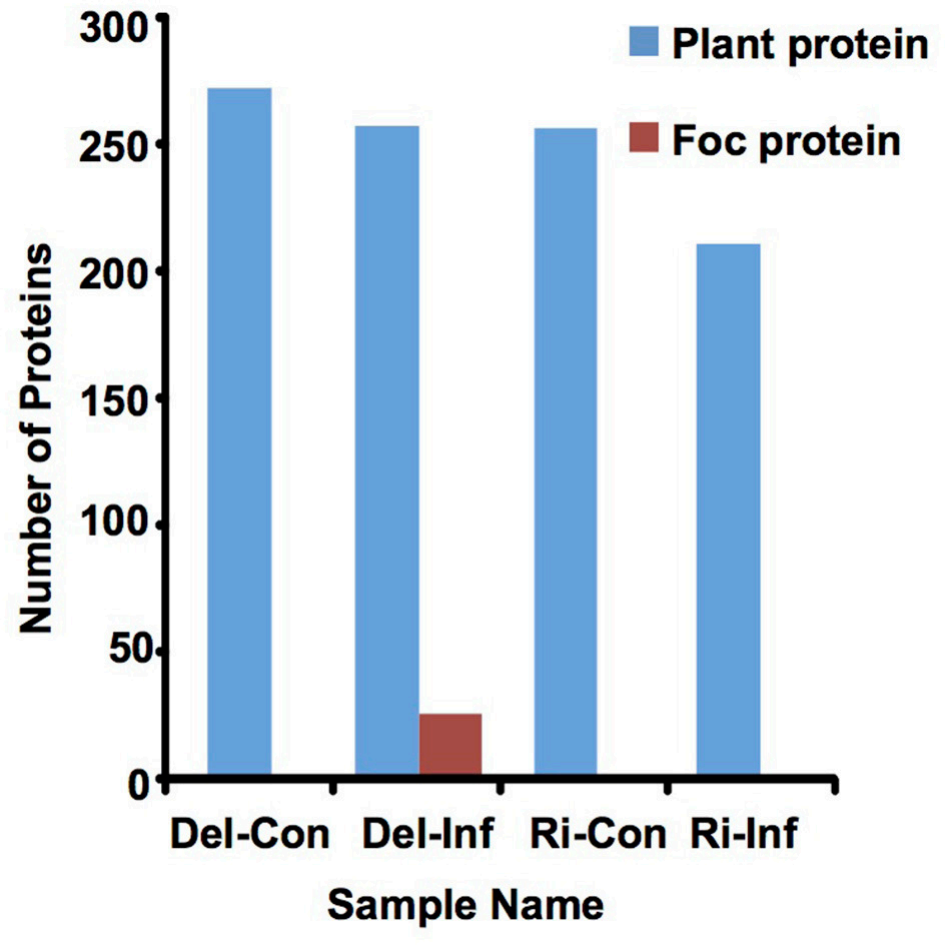
**Number of proteins detected in xylem sap by LC-MS/MS analysis**.

In solution digestion was carried out to make a detailed investigation of protein content in each xylem sap sample, collected from the non-infected and infected plants of Delicious and YCR-Rinen (Del-Con, Del-Inf, Ri-Con, and Ri-Inf, respectively). For the xylem sap proteins of the non-infected plants, the shotgun LC-MS/MS proteomics analysis identified 270 and 254 plant proteins from Del-Con and Ri-Con, respectively (Figure 3, Table S1). VENNY 2.0 (http://bioinfogp.cnb.csic.es/tools/venny/index.html) was then used to investigate the overlap of these proteins between the two samples. Among the proteins detected in non-infected samples, 225 proteins were common between Del-Con (83% of 270) and Ri-Con (89% of 254). The difference of the proteins identified in the two non-infected *B. oleracea* xylem sap samples could be due to differences in the cultivars.

The molecular mass (MM) of *B. oleracea* xylem sap plant proteins varied with a range of 7.6 kDa (M4CY57, Uncharacterized protein) to 160.3 kDa (M4CW30, Tyrosine-protein kinase). However, the majority of plant proteins (86~88%) had a MM between 10 and 70 kDa in size (Figure S1), which coincided with band patterns observed in one-dimensional SDS-PAGE (Figure 2). These results indicate *B. oleracea* xylem sap proteins mainly consist of relatively small sized proteins.

**Figure 4:**
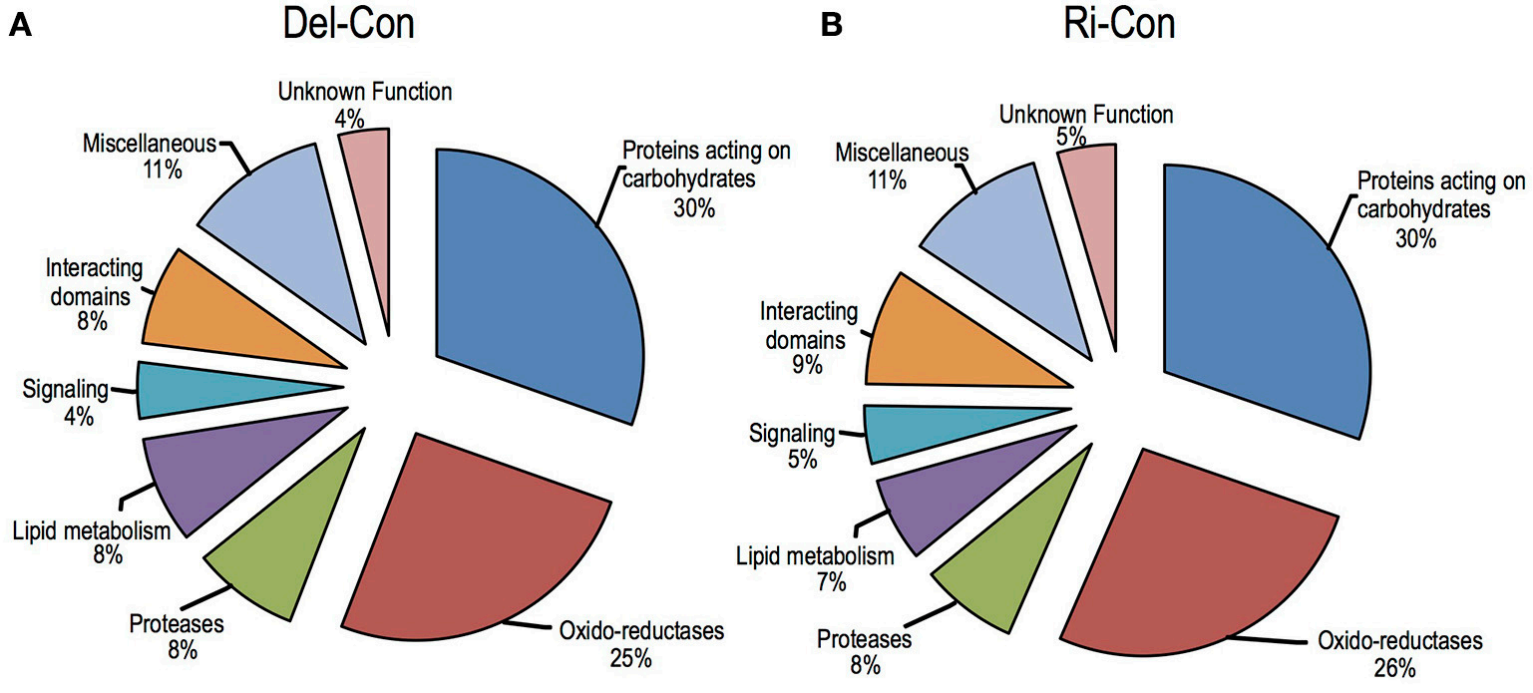
**Functional classification of the secreted plant proteins detected in non-infected *B. oleracea***. **(A)** non-infected Delicious (Del-Con); **(B)** non-infected YCR-Rinen (Ri-Con).

Sub-cellular localization of proteins identified in healthy samples was predicted by bioinformatics software and the proteins were classified into (1) predicted intracellular proteins (about 20% of total) that were devoid of signal peptide and (2) secreted proteins (about 80% of total). The secreted proteins were further sorted into eight functional classes based on the previous study of *A. thaliana* cell wall proteins (Jamet et al., 2006) and *B. oleracea* xylem sap proteome (Ligat et al., 2011); proteins acting on carbohydrates (31~30%, differ between Delicious and Rinen), oxido-reductase (27~28%, as above), proteases (8~7%), proteins involved in lipid metabolism (8~6%, as above), proteins involved in signaling (4%), proteins with domains interacting with carbohydrates or proteins (7~9%, as above), proteins with diverse functions (11%), and proteins with yet unknown function (4%; Figure 4). The main group in proteins acting on carbohydrates was glycoside hydrolase; oxido-reductases mainly consisting of FAD-binding, peroxidase, and plastocyanin-like family; almost all the proteins in proteases group were peptidases; LTPs were the main content of lipid metabolism; all the proteins in signaling group were FAS1 (fasciclin-like) domain proteins, which may function in cell communication and adhesion (Johnson et al., 2003).

**Figure 5:**
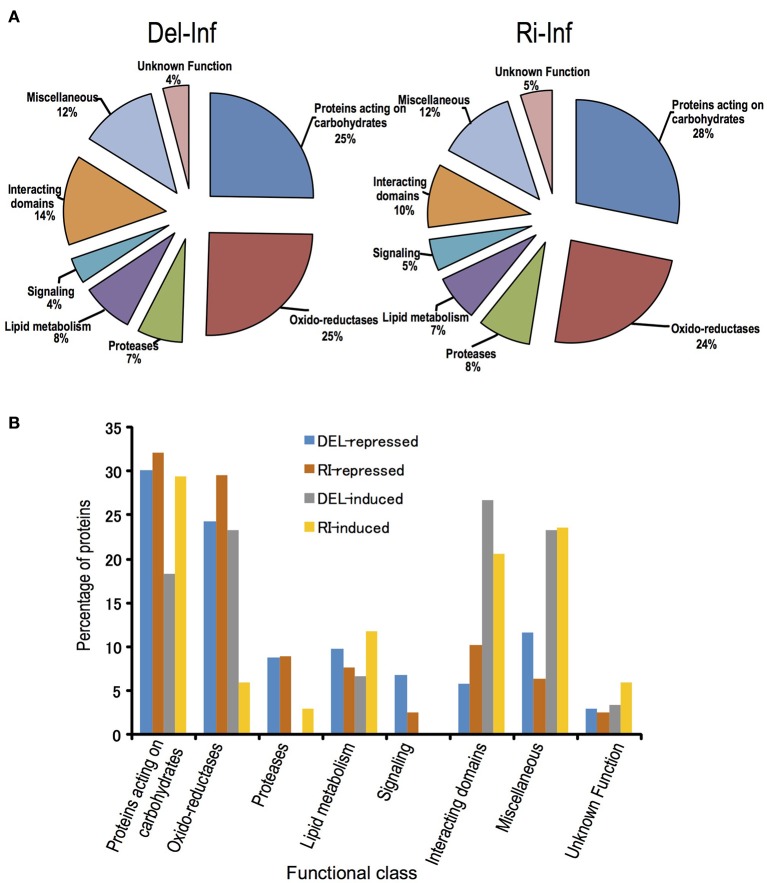
**Functional classification of the secreted proteins detected in the Foc-infected *B. oleracea*, and proteins induced or repressed by Foc-infection. (A)** Functional class was classified according to Ligat et al. (2011). **(B)** Protein induced or repressed in Delicious or YCR-Rinen is indicated by Del-induced, Del-repressed, Ri-induced, and Ri-repressed, respectively. Percentage of protein is calculated by dividing the number of each item with total induced or repressed protein number in each cultivar.

For the xylem sap proteins of the infected plants (Del-Inf and Ri-Inf), 255 plant proteins were identified from Delicious (Del-Inf), and 209 were identified from YCR-Rinen (Ri-Inf), (Figure 3, Data Sheet 1). Foc proteins were only identified from the xylem sap collected from susceptible Delicious that was infected by Foc (later mentioned). The relatively small number of proteins detected in Foc-infected YCR-Rinen (resistant) may indicate a resistant reaction, because tyloses are expected to induce in resistant plants to occlude the xylem, and thus limit the pathogen's growth as well as the protein transport in plant xylem (reviewed in Yadeta and Thomma, 2013). The functional classification of the secreted proteins detected in the Foc-infected *B. oleracea* is shown in Figure 5A. This result enables us to analyze changes that have occurred between healthy and infected samples, as follows.

### Identification of differential and unique proteins in *B. oleracea* during foc infection

Profile comparisons between Foc-infected and non-infected within each cultivar, i.e., Del-Con vs. Del- Inf as well as Ri-Con vs. Ri-Inf, were carried out (Figure 5, Data Sheet 2). The results are as follows. First, the total number of proteins repressed after infection was larger than induced in both the resistant and susceptible groups. In YCR-Rinen (resistant), 112 secreted proteins (among 155 proteins, including 43 intercellular proteins) were either unique or show a fold change > = 2, including 78 repressed and 34 induced. On the other hand, in Delicious (susceptible), the 164 secreted proteins (among 204 proteins, including 40 intercellular proteins) had a fold change > = 2 in Delicious (susceptible), including 103 repressed and 61 induced (Data Sheet 2). The greater number of repressed proteins may indicate a suppressed transporting/metabolism in xylem in both Foc-resistant and -susceptible system. Indeed, no signaling related protein (FAS1 domain proteins) was induced in either of the reactions (Figure 5). The suppressed metabolism in xylem is considered to coincide with the symptom of stunned and retard growth triggered by Foc. Second, similar percentage of proteins acting on carbohydrates was induced or suppressed in both the resistant and susceptible reactions, which demonstrate a role of these proteins in Foc resistance (Figure 5). Third, lipid metabolism related proteins, including the so-called PR-14 (lipid-transfer proteins, LTP) that contribute to plant defense response, also showed up- and down-regulation in similar percentages in both the resistant and susceptible systems. Additionally, in the groups of proteins with interacting domains and miscellaneous proteins having diverse functions, a higher number of proteins were induced by Foc-infection (Figure 5, Data Sheet 2). This is mainly due to the induction of lectins, leucine-rich repeat (LRR) and thaumatins by Foc-infection. Furthermore, although both up- and down-regulated oxido-reductases and proteases were detected in the susceptible reaction, only a few oxido-reductases were induced in resistant system (Figure 5). Such a differential response on oxido-reductases indicates that the induced oxido-reductases were considered to be related to symptom development in susceptible plant.

### Foc proteins identified from the xylem sap of the infected delicious

Whole genome sequencing for Foc was carried out and generated a Cong: 1-1 protein sequences database with 17,009 predicted proteins (manuscript in preparation) using for Foc protein identification in this study. Foc proteins were only identified from the xylem sap collected from susceptible Delicious that was infected by Foc (Del-Inf). In total, 25 predicted Foc proteins were detected in Del-Inf (Table 1). The MM of Foc proteins identified varied from 12.4 kDa (P11242) to 104.8 kDa (P01134). Twenty-four proteins (96% of total number proteins detected) were distributed among 10~70 kDa in size (Figure S1, Foc protein), and P01134 is the only Foc protein that is larger than 70 kDa identified in our study.

**Table 1:** **Foc proteins identified in the xylem of Foc-infected *B. oleracea* cv. Delicious**.

**No**.	**Accession number^a^**	**Description^b^**	**Mass (kDa)**	**SP^c^**	**Length (aa)^d^**	**Cys Number**	**Score/Coverage^e^**
1	P00041/contig_75_2	Carboxylesterase, type B	60.9	N	558	5(0.90%)	821/43.4
2	P01134/contig_644_31	Ferric reductase Fre2p	104.8	Y	932	16(1.72%)	305/5.6
3	**P01592/contig_663_2**	Putative glycosidase crf1	29.3	Y	274	5(1.82%)	350/19
4	**P03403/contig_739_59**	**Protein SnodProt1**	14.6	Y	139	4(2.88%)	636/70.5
5	**P04292/contig_761_13**	Endoglucanase c	32.3	Y	294	3(1.02%)	228/26.5
6	P05132/contig_785_69	Valacyclovir hydrolase	32.0	N	290	1(0.34%)	397/39.7
7	P08654/contig_907_28	Alcohol dehydrogenase	65.3	Y/M	609	3(0.49%)	235/20.9
8	P10310/contig_989_8	Putative serine-rich protein	41.3	Y	388	8(2.06%)	116/5.7
9	P10456/contig_995_21	**Endo-1,4-beta-xylanase C**	35.7	Y	328	2(0.61%)	211/23.2
10	P11164/contig_1045_2	Chitinase	47.4	Y	432	13(3.01%)	130/19.9
11	P11220/contig_1051_3	BNR/Asp-box repeat domain protein	41.3	Y	379	3(0.79%)	1045/52.0
12	**P11242/contig_1054_1**	Hypothetical protein	12.4	Y	117	2(1.71%)	259/50.4
13	**P11311/contig_1060_12**	**LysM domain-containing protein**	28.4	Y/M	265	12(4.53%)	167/11.7
14	P11347/contig_1063_1	Secreted oxidoreductase ORX1-like protein	65.2	Y	610	3(0.49%)	688/37.1
15	**P13298/contig_1456_1**	Hypothetical protein	19.1	Y	170	2(1.18%)	217/40.6
16	P13299/contig_1456_2	Hypothetical protein	25.2	N	228	10(4.39%)	927/41.7
17	P13310/contig_1464_1	Hatching enzyme	38.2	Y	338	6(1.78%)	511/47.9
18	**P13373/contig_1509_1**	Hypothetical protein	29.1	Y	263	8(3.04%)	464/38.0
19	**P14728/contig_2465_1**	**Foc-SIX4**	27.3	Y	252	6(2.38%)	296/35.3
20	P14743/contig_2555_1	Hatching enzyme	45.3	Y	402	12(2.99%)	567/46.5
21	P15123/contig_3892_1	FAD binding domain-containing protein	55.0	Y/M	497	2(0.40%)	328/19.9
22	P15632/contig_3990_11	Chitinase A1	45.6	Y	419	5(1.19%)	563/28.6
23	**P15981/contig_4126_1**	**Foc-SIX1**	30.8	Y	279	9(3.23%)	369/52.7
24	P16246/contig_4338_1	**Endo-1,4-beta-xylanase**	25.1	Y	232	0(0.00%)	202/32.3
25	**P16923/contig_5980_1**	hypothetical protein	18.6	Y	167	2(1.20%)	241/74.9

a *Serial number/contig number of Foc Cong: 1-1 genome database used in this study. The numbers given in bold indicate a small (less than 300 amino acids) cysteine-contained secreted protein*.

b *Genes were annotated by NCBI blastp. Descriptions of the functional/putative functional protein item were used. Descriptions given in bold were discussed in Discussion. A detail result of protein BLAST consults Table S4*.

c *SP indicates a predicted signal peptide denoting secretion. The signal peptide was predicted using SignalP (http://www.cbs.dtu.dk/services/SignalP/) and TargetP (http://www.cbs.dtu.dk/services/TargetP/). “Y” and “N” indicate whether or not the sequence contains a signal peptide in secretory pathway. When both predictions are consistent, only the SignalP result is shown. When it is not the case, both predictions are shown. “M” indicates that TargetP predicted a mitochondrial targeting peptide*.

d *Length given in amino acid number is count from the full length of predicted peptide sequence generated from Foc genome information*.

e *Number before the slash indicates the score and after the slash indicated the coverage (given in percentage) evaluated by MASCOT*.

Since small cysteine-rich proteins secreted by phytopathogenic fungi into their host are implicated as effectors involved in disease development and R-gene-mediated resistance (Reviewed in Rep, 2005; Stergiopoulos and De Wit, 2009), we predicted the sub-cellular localization and analyzed total protein length as well as the cysteine content calculation (cysteine number divided by total protein length) of identified proteins (Table 1). Of the 25 proteins, 22 were predicted as secreted protein by SingalP or TargetP, while the remaining three (P00041, P05132, P13299) were predicted as intracellular proteins. Eleven of the twenty-three secreted proteins were regarded as small proteins with a length less than 300 amino acids and one of them (P16246) contains no cysteine. That is, we detected 10 small secreted cysteine-containing Foc proteins during the interaction between Foc and *B. oleracea*.

To annotate the identified Foc proteins, we performed similarity search of the predicted protein sequences using the NCBI protein BLAST tool. Based on the alignment list generated by the NCBI blast tool, we selected the top match from in the BLAST results for annotation (Table S4). Since most of them were hypothetical proteins, a functional/ putative functional protein item in each BLAST result with max score >150 and query cover >60% was also selected as reference (Table S4) and list in the Table 1 as description. Among the 25 identified Foc proteins, five of them (P11242, P13298, P13299, P13373, and P16923) have no reliable functional/ putative functional protein item in the BLAST result (Table 1, Table S4). Most of the annotated proteins were enzymatic active protein, such as chitinase (P11164), putative oxidoreductase (P11347), and xylanase (P10456 and P16246).

**Figure 6:**
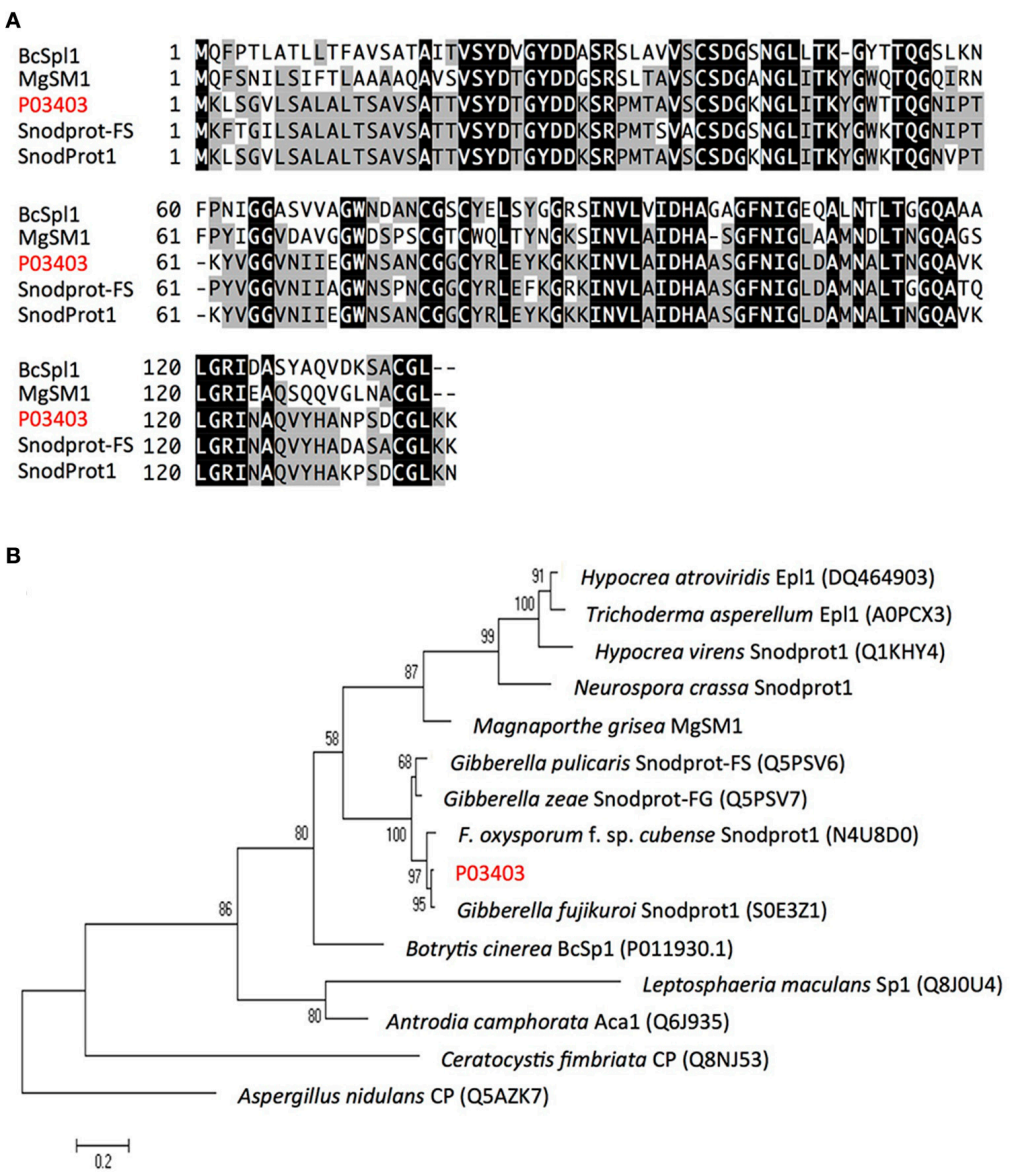
**Alignment and phylogenetic tree analysis of Foc protein P03403. (A)** Alignment of P03403 (shown red letters) with other known fungal proteins using ClustalW2 (http://www.ebi.ac.uk/Tools/msa/clustalw2/). Fungal proteins used for alignment include Snodprot1 of *Gibberella fujikuroi*, Snodprot-FS of *Gibberella pulicaris*, MgSM1 of *Magnaporthe grisea* and BcSpl1 of *Botrytis cinerea*. A predicated signal peptide is indicated by dotted line based on the result from SingalP (http://www.cbs.dtu.dk/services/SignalP/). Tryptic peptides identified in LC-MS/MS analysis was underlined. **(B**) Neighbor-joining tree analysis of P03403 with other proteins from various fungal species. Neighbor-joining tree was constructed using MEGA version 5. Numbers on nodes represent bootstrap values estimated from 1000 replications of the data set. The bar indicates a phylogenetic distance. *Aspergillus nidulans* CP (Q5AZK7) was used as outgroup.

P14728 and P15981 show 100% identity with the reported Foc-*Six4* and Foc-*Six1* gene, respectively. Foc-*Six4* has been demonstrated as a virulence factor toward *A. thaliana* and cabbage (Thatcher et al., 2012; Kashiwa et al., 2013). However, the expression of Foc-*Six1* during infection and its effector function has yet to be confirmed. Our study indicated a secretion of Foc-*Six1* into susceptible plant xylem during Foc-infection. In addition, P11311 containing two Lysin motifs (LysMs), was determined as a LysM-contains protein (Figure S2); P03403 contains a cerato-platanin (CP) domain and its predicted amino acid sequence shares 91.9% similarity with some reported CP proteins including MgSM1 (a CP protein from *Magnaporthe grisea*), thus determined as a CP protein (Figure 6).

## Discussion

### Determining the time-point for harvesting the samples

In this study, we demonstrated that the mycelia penetrate the surface of root apex and LRP, traverse cortical tissues and reach the vascular cylinder at 7 dpi in the susceptible cultivar. After that the mycelia quickly proliferated in the root in the susceptible plants. In contrast, in the resistant plant, the mycelia were restricted at the point of infection, such as the root apex. This observation is in agreement with the result reported by Li et al. (2015), who performed microscopical analysis using the GFP-expressing strain of Foc to the 2–3 leaf stage of cabbage. Li et al. (2015) reported that the Foc-colonization in the root of the susceptible plants reached a maximum at the 11 dpi. Therefore, the time point at 12 dpi is thought to be suitable for the collection of xylem sap.

### Plant proteins in the xylem sap of the non-infected *B. oleracea*

Previous studies examined the xylem sap through a precise gel-dependent separation and digestion (Rep et al., 2002; Buhtz et al., 2004; Houterman et al., 2007; Floerl et al., 2008). However, the number of proteins identified was limited because not all of the proteins could be resolved by gel electrophoresis and further analysis for the detected proteins patterns (one-dimensional) or spots (two-dimensional) would be time and labor consuming. For example, Kehr et al. (2005) carried out proteome analysis for *B. napus* xylem sap based on 2-dimensional analysis and identified 69 proteins. Thereafter, application of high-throughput LC-MS/MS improved resolution for proteins and thus increased the number of proteins identified. A representation of xylem sap proteome analysis is Ligat et al. (2011) who reported 189 proteins in *B. oleracea* xylem. Our study adopted in-solution digestion, which yielded about 200 proteins in *B. oleracea* xylem. Despite the different techniques and databases for protein identification, close similarity between *A. thaliana* and *Brassica* genes allowed the comparison of our study with previous studies (Table S1). For example, 32 and 150 unique *A. thaliana* homologous genes were reported in the study of Kehr et al. (2005) and Ligat et al. (2011), respectively. About 84% (27 of 32) unique *A. thaliana* genes homologous with the *B. napus* protein identified by Kehr et al. (2005) were present in our study, demonstrating a relationship between *B. oleracea* and *B. napus* that *B. oleracea* (C genome) is a progenitor species of *B. napus* (AC genome) (Table S1). Compared with the previous report by Ligat et al. (2011), about 75% of the *A. thaliana* homologous genes (112 of 150 unique ID) identified in Ligat et al. (2011) are present in our study (Table S1). There appears to be no remarkable differences in protein families identified between the studies, however there are variations in the constitution and protein numbers in each protein family observed between Ligat et al. (2011) and our study. The difference may result from cultivars, sampling time points or the different technologies used in the bioinformatic analysis.

It has been suggested that xylem sap protein composition is conserved among species, since some of the most abundant proteins are commonly present within plant xylem of various cultivars (Buhtz et al., 2004; Dafoe and Constabel, 2009). Many of these proteins, such as PRs, oxido-reductases and proteases, could be induced by the stress of the unavoidable decapitation step during xylem sap collection (Kehr et al., 2005; Alvarez et al., 2006; Djordjevic et al., 2007; Aki et al., 2008; Floerl et al., 2008; Dafoe and Constabel, 2009; Ligat et al., 2011; Zhang et al., 2015). In the present study, these proteins were also detected in *B. oleracea* xylem sap in both the non-infected and Foc-infected plants (Data Sheet 1).

### Comparison of the xylem sap proteins in the non-infected with foc-infected plants

Our study suggests a regulation of xylem sap proteins driven by Foc-infection (Figure 5). It is well known that protein-carbohydrate interactions play an important role in the recognition of pathogens. Carbohydrate structures, which are either present at the surface of the invading pathogen cell or released from degraded plant cell wall damaged by pathogen entry, are the main part of the pathogen/damage-associated molecular patterns (P/ DAMPs) perceived in the plant and triggered by the innate immunity response of the plant (reviewed in Lannoo and Damme, 2014). In our study, the large portion of up- and down-regulated proteins acting on carbohydrates in both resistance and susceptible system also suggests complex and intense protein-carbohydrate interactions in *B. oleracea* xylem sap driven by Foc-infection (Figure 5, Data Sheet 2). Among these carbohydrate interacting proteins, β-1,3-glucanases and chitinase have been reported to contribute to biotic stress response and are ubiquitous in plant xylem (Punja and Zhang, 1993; Buhtz et al., 2004; Ligat et al., 2011; Ahmed et al., 2012). Rep et al. (2002) reported an accumulation of β-1,3-glucanases and chitinase in tomato xylem sap after Fol-infection. They were also up-regulated in *B. napus* upon *V. longisporum* infection (Floerl et al., 2008). In our study, Foc infection in both resistant and susceptible plants activated chitinases and β-1,3-glucanase. Although they may be involved in PTI (PAMP-triggered immunity) in *B. oleracea*, such a defense mechanism was not strong enough in the susceptible plants, such that Foc successfully persisted in parasitic growth. However, in the resistant plant, certain effector(s) triggered an R-gene (*FocBo1*, later mentioned) mediated resistance reaction (ETI) that restricts Foc growth.

Our previous study identified the *Foc-Bo1*, a candidate gene, conferring *Fusarium* resistance to *B. oleracea* (Shimizu et al., 2015). Its homologous gene in *B. rapa*, named *Foc-Br1*, was also identified which was shown to be a candidate of Foc resistant gene (Shimizu et al., 2014). Since both of them are NBS-LRR type resistant genes, they are considered as the main R-gene involved in mediated resistance against Foc. We did not detect the *Foc-Bo1* protein in the xylem sap of YCR-Rinen, a cultivar that contains the *Foc-Bo1* gene (Shimizu et al., 2015). In the tomato-Fol system, the nuclear migration of *Avr2* from Fol is required to activate the tomato resistance protein *I-2*, which triggers cell death as a consequence of recognition of *Avr2* (Ma et al., 2013). It is therefore plausible that the R-gene mediated resistance against Foc is also an in-cell reaction. That is to say, *FocBo1* perceives Foc in the cell and immediately triggers HR to limit the further growth of Foc. The effective programmed cell death limits the transport of *Foc-Bo1* protein in xylem sap of *B. oleracea*.

Different from the group of proteins acting on carbohydrates which showed a similar number of up- and down- regulated proteins due to Foc-infection, a larger portion of proteins with interacting domains and miscellaneous proteins having diverse functions were induced after Foc-infection. In the interacting domains group, the induced proteins were mainly leucine-rich repeat (LRR) protein and legume lectin (Table S2). The LRR domain plays an important role in direct/indirect recognition of pathogenic effector proteins, and lectin domains are implicated in the recognition of carbohydrate structures that are perceived as “danger” molecules (Reviewed in Lannoo and Damme, 2014). In the miscellaneous protein group, the induction mainly comes from Thaumatin. Thaumatin (PR-5) is a kind of sweet protein that was first reported in *Thaumatococcus danielli*. Since then, studies have identified Thaumatin-like proteins (TLPs) from many different plants and classified these TLPs into PR-5 proteins, based on their ability to respond to biotic and abiotic stress (Van Loon et al., 2006). The anti-fungal activity of TLPs against plant pathogenic *F. oxysporum* has been demonstrated in *A. thaliana* (Hu and Reddy, 1997), French bean (Ye et al., 1999), tomato (Rep et al., 2002), and cotton (Munis et al., 2010). Our study showed that thaumatin proteins were induced at a similar level in both resistance and susceptible systems, with the exception of M4EA13 and M4CVH7 that was specifically accumulated in YCR-Rinen and Delicious, respectively (Table S3). The fact that Foc colonization proceeds in Delicious in spite of the accumulation of PR-5 proteins implies that Foc could avoid or resist potential anti-fungal activity of these TLPs. Further study is required to determine whether the M4EA13 produced in YCR-Rinen contributes specifically against Foc infection, or the accumulation of thaumatins in Delicious occurs too late (than YCR-Rinen) to prevent Foc infection.

Interestingly, oxido-reductases are repressed in both cultivars but induced only in the susceptible system (Figure 5). Oxidative bursting is one of the earliest responses in the root against *F. oxysporum* infection (Plancot et al., 2013). Previous studies suggest reactive oxygen species (ROS) act as signaling molecules thus contributing to plant defenses (Torres, 2010). However, it has been recently suggested that ROS may also promote disease development of some pathogens. Lyons et al. (2015) reported that PRX33, which is required for ROS formation and MAMP-triggered ROS production, promotes susceptibility to *F. oxysporum*. Therefore, the induction of oxido-reductases in this study may relate to the development of Fusarium-wilt.

### Fungal proteins detected in *B. oleracea* xylem

Identification of *Fusarium* proteins in plant xylem has been thoroughly carried out in tomato-Fol system (Rep et al., 2002; Houterman et al., 2007). However, due to the lack of genome information of Fol at that time, identification of Fol original proteins was dependent on the information of homologous peptides of other species. This hindered previous identification work and limited the number of proteins that could be identified. To facilitate the accuracy of protein identification, whole genome sequencing of Foc was carried out and a Foc protein database with 17,009 predicated proteins was generated in this study. Finally, we detected 25 Foc-proteins in infected *B. oleracea* xylem, which strongly indicated these proteins play some roles during infection. Thus, functions of these proteins are also of great interest. Among the detected proteins, 22 were predicted to have a signal peptide that connotes active secretion. Given small proteins secreted by plant-pathogenic fungi in the hosts have been implicated in disease symptom development as well as in R-gene mediated disease resistance (Reviewed in Rep, 2005), proteins containing less than 300 amino acids are of great interest. Since the cloning of the first Six gene (*Six1*/*Avr3*) from Fol, 14 Fol-*Six* effector candidate proteins have been identified (Rep et al., 2004; Schmidt et al., 2013). *Six1, Six4, Six8*, and *Six9* homologs have been detected in Foc but only *Six4* has been cloned and confirmed as a virulence factor to *A. thaliana* and cabbage (Thatcher et al., 2012; Kashiwa et al., 2013). Our study detected the reported Foc-*Six4* protein as well as the not yet characterized Foc-*Six1* protein in infected xylem sap. Foc-*Six1* shares 80% amino acid similarity with Fol-*Six1* which is required for full virulence and recognized by the *I-3* resistance gene in tomato (Rep et al., 2004, 2005; Thatcher et al., 2012). However, the expression and function of Foc*-Six1* during infection has not been reported. The detection of Foc-*Six1* proteins in infected cabbage strongly indicates the expression of Foc-*Six1* during infection. Further studies should focus on the function of Foc- *Six1*.

Protein annotation indicates P03403 is a Snodprot protein that has similarity with the reported cerato-platanin (CP) family proteins, such as Snodprot1 of *Neurospora crassa* (Q9C2Q5), Snodprot-FG (Q5PSV6) of *Gibberella zeae*, MgSM1 of *Magnaporthe grisea*, and BcSpl1 in *Botrytis cinerea* (Jeong et al., 2007; Yang et al., 2009; Frias et al., 2011; Figure 6). To date, CP proteins have only been reported in fungi, are widespread within fungi, conserved in structure, and abundantly secreted. Additionally, CP proteins have been reported to act as pathogen-associated molecular patterns (PAMPs). Ectopic expression of *MgSM1* gene in *A. thaliana* activates plant defense response, resulting in broad-spectrum resistance against different fungal and bacterial pathogens (Yang et al., 2009). *A. thaliana* lacking the *BAK1*gene of the PAMP signaling pathway prevented the induction of necrosis in this mutant by *BcSpl1* (Frias et al., 2011). Furthermore, some of the CP proteins have been shown to act as virulence factors, i.e., knockout of *BcSpl1* in *B. cinerea* and *MSP1* in *M. grisea* showed reduced virulence to their host plant, respectively (Jeong et al., 2007; Frias et al., 2011). Recent studies indicate some MpCPs (CP proteins from *Moniliophthora perniciosa*) are able to bind chitin with high affinity, and consequently, this strong affinity for chitin could sequester the excitation of the plant immune system elicited by fungal chitin fragments (De O Barsottini et al., 2013; Baccelli et al., 2014). To our knowledge, the function of CP proteins has not been reported in *F. oxysporum* species. The detection of P03403 protein strongly indicates it plays a role in Fusarium-host interaction. Gene knockout experiments are ongoing to confirm this hypothesis.

In addition to CP proteins, LysM (Lysin motif)-containing effectors are also widespread in fungi and are proposed to contribute to virulence by sequestration of chitin oligosaccharides released from fungus during infection, thereby blocking the activation of host chitin receptors (Reviewed in De Jonge and Thomma, 2009). For example, the secreted LysM Protein1 (*Slp1*, required for full virulence toward rice) from *M. oryzae* is accumulated at the interface between the fungal cell wall and the rice plasma membrane. It competes with the chitin elicitor binding protein (CEBiP) for the binding of chitin oligosaccharides and it is therefore proposed to suppress chitin-induced plant immune response, facilitating virulence (Mentlak et al., 2012). Ecp6 from *Cladosporium fulvum* is also a famous LysM-containing effector, which suppress chitin-triggered immunity through intra-chain LysM dimerization and/or through binding to chitin oligomers thereby physically blocking host immune receptor dimerization (De Jonge et al., 2010; Sánchez-Vallet et al., 2013). In our study, the detected xylem secreted Foc protein P11311, contains two LysMs. Thus, this result generates a question for the function of LysM-containing protein during Foc infection (Figure S2). Further studies should reveal whether, and how, perturbation of chitin-triggered immunity by LysM occurs in the interaction of Foc with *B. oleracea*.

The only cysteine-free small secreted protein is P16246 which has 94.2% similarity with FGSG_03624, an endo-1,4-β-xylanase of *Fusarium graminearum* (Sella et al., 2013). In addition to P16246, another endo-1,4-β-xylanase, P10456, was also detected in this study. P10456 is 328 amino acids in length and has 99.2% identity with xyl2 of Fol (GenBank accession No. AF052583, Ruiz-Roldan et al., 1999). Endo-1,4-β-xylanase are produced by many plant pathogenic fungi and are likely to be involved in the degradation of cell walls during host colonization thereby facilitating infection/pathogenicity (Walton, 1994; Kikot et al., 2009). However, xyl2 is only expressed in the final stages of tomato wilt, and thus may associate with saprophytic growth (Ruiz-Roldan et al., 1999). It was suggested recently that xylanase contributes to virulence not by enzymatic activity but also with its necrotizing activity (Enkerli et al., 1999; Noda et al., 2010). Both of the FGSG_03624 (Sella et al., 2013) and the P16246 (our study) share similarity with the amino acids in regards to necrosis elicitation. However, whether P16246 has a similar ability to FGSG_03624 to induce necrosis in infected tissue remains elusive.

In conclusion, to our knowledge, the present study is the first report regarding to identification of protein changes driven by Foc-infection in *B. oleracea* xylem sap in both the resistant and susceptible systems. The large number of up- and down-regulated proteins acting on carbohydrate as well as the induced LRR and legume lectin domain proteins suggests a complex recognition of Foc in *B. oleracea*. In addition, the induced oxido-reductases in the susceptible reaction may indicate a contribution of ROS to disease development. Importantly, our study also reported 25 predicted Foc proteins in susceptible plant xylem sap infected by Foc. Eleven of them are small (less than 300 amino acid in length) secreted Foc proteins in the infected *B. oleracea* with 10 of them containing cysteine. These Foc-proteins are of great interest because they are good candidates for virulence and/or avirulence factors. Thus, the present study provides important resources for study on Foc effector proteins as well as the mechanisms of the interaction between Foc and *B. oleracea*.

## Author contributions

ZP contributed to the study as the first author. YI, KK, and YK partly conducted LC-MS/MS analysis. SF conducted Illumina sequence. AT, MS, DS, and SN conducted bioinformatics analysis. YS took charge of fungal culture. RF conducted experimental design and statistical analysis. HH contributed to proteome experimental design. KO partly contributed to writing manuscript, provided funding, and managed the whole project.

## Funding

The support provided by China Scholarship Council (CSC) during a visit of ZP to Niigata University is deeply acknowledged. This work was supported by the Programme for Promotion of Basic and Applied Researches for Innovations in Bio-oriented Industry to KO.

### Conflict of interest statement

The authors declare that the research was conducted in the absence of any commercial or financial relationships that could be construed as a potential conflict of interest.
