# Supplementary material for: Changes in the Proteome of Xylem Sap in Brassica oleracea in Response to Fusarium oxysporum Stress
Source: Front Plant Sci. 2016 Feb 1;7:31. doi: 10.3389/fpls.2016.00031 (PMC4734173; doi:10.3389/fpls.2016.00031)
Supplement: Supplementary file 1 [file Presentation1.PDF]

Table S1. Plant proteins identified by LC-MS/MS in the xylem sap of non-infected Delicious and YCR-Rinen.

No.[a]	Access Number[b]	Gene Name	Homologous *A.thaliana*[c]	Description[d]	Mass (kDa)	SP[e]
**Proteins acting on carbohydrates**						
26	M4CAZ7	Bra001376	***AT3G10740***	Alpha-L-arabinofuranosidase	60.3	N/S
319	M4EZ98	Bra034141	***AT3G10740***	Alpha-L-arabinofuranosidase	74.4	N/S
3	A4USG1		***AT3G57260***	Beta-1.3 glucanase	38.9	Y
20	M4C8G5	Bra000493	***AT2G28470***	Beta-galactosidase	92.1	Y
121	M4D638	Bra011946	***AT2G28470***	Beta-galactosidase	91.9	Y
193	M4DRH7	Bra019120	***AT4G26140***	Beta-galactosidase	79.8	Y
239[D]	M4E634	Bra024238	AT5G63810	Beta-galactosidase	82.9	Y
375	Q05K38	CHB4	**AT2G43590**	Chitinase	28.7	Y
14	M4C7Y2	Bra000310	*AT2G43570*	Chitin-binding	30.3	Y
15	M4C7Y7	Bra000315	***AT2G43620***	Chitin-binding	30	Y
54[D]	M4CKN7	Bra004772	***AT2G43620***	Chitin-binding	30.4	Y
323	M4F109	Bra034754	**AT3G12500**	Chitin-binding	34.9	Y
130	M4D874	Bra012684	***AT4G17030***	Expansin 45	27.9	Y
269	M4EH26	Bra028091	AT2G18660	Expansin 45	14	Y
120	M4D5Z1	Bra011899	AT4G38400	Expansin 45	29.1	Y
366	M4FG80	Bra040107	***AT4G17030***	Expansin 45	27.8	Y
31	M4CDJ0	Bra002271	***AT3G21160***	Glycoside hydrolase	30.1	N/S
33	M4CDR5	Bra002346	AT5G20950	Glycoside hydrolase	66.4	Y
42	M4CFE5	Bra002927	***AT5G55180***	Glycoside hydrolase	49.8	Y
44[D]	M4CG79	Bra003212	***AT3G55430***	Glycoside hydrolase	48.5	Y
45	M4CGE0	Bra003273	***AT3G57240***	Glycoside hydrolase	37.6	Y
278	M4EIP6	Bra028661	**AT5G08370**	Glycoside hydrolase	43.9	Y
357	M4FCX5	Bra038945	**AT5G34940**	Glycoside hydrolase family 79	59.8	Y
57[D]	M4CKU9	Bra004835	**AT2G44450**	Glycoside hydrolase, family 1	56.5	Y
166[R]	M4DIE9	Bra016276	AT1G26560	Glycoside hydrolase, family 1	57	Y
176	M4DM78	Bra017610	AT4G33810	Glycoside hydrolase, family 10	52.6	Y
350	M4FAH4	Bra038088	**AT4G15210**	Glycoside hydrolase, family 14	56.1	N/S
35	M4CET7	Bra002719	***AT5G57560***	Glycoside hydrolase, family 16	32	Y
89	M4CX59	Bra008806	**AT4G25810**	Glycoside hydrolase, family 16	32.1	Y
107	M4D3X5	Bra011179	***AT4G30270***	Glycoside hydrolase, family 16	31.8	Y
207	M4DV89	Bra020433	***AT5G57560***	Glycoside hydrolase, family 16	29.6	Y
236[R]	M4E5N5	Bra024089	***AT4G30270***	Glycoside hydrolase, family 16	31.6	Y
197	M4DSC3	Bra019416	AT3G44990	Glycoside hydrolase, family 16	33.1	Y/M
34	M4CEP2	Bra002673	AT5G58090	Glycoside hydrolase, family 17	52.5	Y
74	M4CSG9	Bra007161	***AT3G55430***	Glycoside hydrolase, family 17	48.5	Y
75	M4CSH0	Bra007162	***AT3G55430***	Glycoside hydrolase, family 17	48.6	Y
78	M4CSX3	Bra007315	***AT3G57260***	Glycoside hydrolase, family 17	36.2	Y
115	M4D4Y0	Bra011537	***AT4G34480***	Glycoside hydrolase, family 17	53.2	Y
106[R]	M4D3P6	Bra011100	***AT4G29360***	Glycoside hydrolase, family 17	54	Y
179	M4DMC6	Bra017659	***AT4G34480***	Glycoside hydrolase, family 17	58.5	Y

(Continued) Table S1.

No.[a]	Access Number[b]	Gene Name	Homologous *A.thaliana*[c]	Description[d]	Mass (kDa)	SP[e]
280	M4EJM2	Bra028988	***AT5G55180***	Glycoside hydrolase, family 17	62	Y
315	M4EXL2	Bra033549	AT4G16260	Glycoside hydrolase, family 17	39.2	Y
316	M4EXV2	Bra033641	**AT5G42720**	Glycoside hydrolase, family 17	46.6	Y
328	M4F3B1	Bra035561	***AT5G55180***	Glycoside hydrolase, family 17	49.2	Y
363	M4FF85	Bra039757	AT1G66250	Glycoside hydrolase, family 17	54.2	Y
257	M4ECG0	Bra026469	**AT5G24090**	Glycoside hydrolase, family 18	32.9	Y
336[D]	M4F5G0	Bra036316	*AT4G01700*	Glycoside hydrolase, family 19,	31.4	Y
46	M4CH02	Bra003485	**AT3G62110**	Glycoside hydrolase, family 28	50.8	N/S
80	M4CTR5	Bra007609	***AT3G61490***	Glycoside hydrolase, family 28	51.4	Y
136	M4DB37	Bra013697	***AT4G23500***	Glycoside hydrolase, family 28	54.4	Y
167[D]	M4DIL8	Bra016345	AT1G23460	Glycoside hydrolase, family 28	50.6	N/S
195	M4DS06	Bra019299	***AT4G23500***	Glycoside hydrolase, family 28	54.4	Y
272[R]	M4EI96	Bra028511	**AT5G41870**	Glycoside hydrolase, family 28	48.1	Y
92	M4CXS6	Bra009023	**AT5G10560**	Glycoside hydrolase, family 3	86.3	Y
244	M4E6A7	Bra024311	***AT5G64570***	Glycoside hydrolase, family 3	84.1	Y/M
300	M4ESY2	Bra031912	***AT5G64570***	Glycoside hydrolase, family 3	83.6	Y/M
348	M4F9L9	Bra037782	***AT5G64570***	Glycoside hydrolase, family 3	84	Y/M
64	M4CPH3	Bra006111	**AT5G11720**	Glycoside hydrolase, family 31	99.5	Y
65	M4CPT8	Bra006227	**AT5G13980**	Glycoside hydrolase, family 38	115.2	Y
52	M4CJW0	Bra004494	AT2G46930	Pectinacetylesterase	45.5	Y
146	M4DD44	Bra014412	AT3G62060	Pectinacetylesterase	51.2	Y
218	M4DZL4	Bra021961	**AT5G45280**	Pectinacetylesterase	42.1	Y
63	M4CP93	Bra006031	***AT5G09760***	Pectinesterase	59.8	Y
276	M4EIK2	Bra028617	***AT5G09760***	Pectinesterase	60.1	Y
126	M4D7A6	Bra012366	AT1G23205	Pectinesterase inhibitor	22.9	Y
188	M4DQA2	Bra018695	***AT1G47960***	Pectinesterase inhibitor	17.4	Y
**Oxido-reductases**						
60	M4CMF4	Bra005392	***AT2G34790***	FAD-binding	59.6	Y
151	M4DEG4	Bra014885	AT1G30710	FAD-binding	59.5	Y
212	M4DWM1	Bra020915	***AT4G20830***	FAD-binding	59.5	Y
217	M4DZI3	Bra021930	***AT2G34790***	FAD-binding	56	Y
227	M4E2X5	Bra023126	AT1G30730	FAD-binding	58.6	Y
228	M4E2Y0	Bra023131	**AT1G30760**	FAD-binding	59.9	Y
273[D]	M4EIB5	Bra028530	AT1G01980	FAD-binding	59.8	Y
354	M4FCH9	Bra038798	***AT4G20830***	FAD-binding	60	Y
355	M4FCI0	Bra038799	***AT4G20830***	FAD-binding	60.1	Y
119[D]	M4D5L4	Bra011771	AT4G37520	Haem peroxidase	35.7	Y
225	M4E2U8	Bra023099	***AT2G37130***	Haem peroxidase	37.1	Y
381	Q39370		**AT5G15350**	Lamin	18.8	Y
21	M4C994	Bra000773	***AT4G12420***	Multicopper oxidase	65.5	Y
86	M4CVJ4	Bra008241	***AT1G76160***	Multicopper oxidase	60.8	Y

(Continued) Table S1.

No.[a]	Access Number[b]	Gene Name	Homologous *A.thaliana*[c]	Description[d]	Mass (kDa)	SP[e]
156	M4DH04	Bra015781	***AT1G76160***	Multicopper oxidase	59.9	Y
185	M4DPN8	Bra018479	***AT4G12420***	Multicopper oxidase	65.5	Y
343[R]	M4F7S6	Bra037137	AT5G66920	Multicopper oxidase	60.5	Y
13	M4C7Q0	Bra000228	***AT2G41480***	Peroxidase	35.7	Y
50[D]	M4CJF9	Bra004343	AT1G68850	Peroxidase	37.1	Y
93	M4CY08	Bra009105	***AT5G05340***	Peroxidase	29.3	Y
95	M4CYC3	Bra009220	AT5G06720	Peroxidase	35.2	N/S
96[R]	M4CYC4	Bra009221	AT5G06730	Peroxidase	37.5	Y
118	M4D5C4	Bra011681	***AT4G36430***	Peroxidase	36.2	Y
143	M4DCI6	Bra014200	*AT1G49570*	Peroxidase	40.1	Y
154	M4DFY1	Bra015404	***AT1G05260***	Peroxidase	35	Y
162[D]	M4DI00	Bra016127	**AT1G71695**	Peroxidase	39.7	Y
173	M4DKA1	Bra016930	***AT2G41480***	Peroxidase	35.4	Y
180	M4DMM6	Bra017761	***AT4G36430***	Peroxidase	36.2	Y
183[D]	M4DNB9	Bra018006	***AT3G49120***	Peroxidase	39	Y
203	M4DU79	Bra020072	***AT5G19890***	Peroxidase	34.8	Y
242	M4E665	Bra024269	**AT5G64120**	Peroxidase	34.7	Y
270[D]	M4EI21	Bra028436	**AT5G39580**	Peroxidase	34.2	Y
279	M4EIZ6	Bra028761	***AT5G05340***	Peroxidase	34	Y
285	M4EMB3	Bra029933	***AT3G49120***	Peroxidase	38.5	Y
288	M4EN71	Bra030241	***AT2G22420***	Peroxidase	36.7	Y
296	M4ER86	Bra031309	AT3G21770	Peroxidase	35.5	Y
302	M4ET04	Bra031934	AT5G64100	Peroxidase	35.6	Y
307	M4EUJ2	Bra032474	***AT1G05260***	Peroxidase	31	Y
327	M4F2D7	Bra035235	**AT4G11290**	Peroxidase	35.3	Y
342	M4F7E7	Bra037007	***AT4G33420***	Peroxidase	33.3	N/S
365	M4FFP6	Bra039920	AT2G18150	Peroxidase	36.4	Y
28	M4CC64	Bra001794	***AT3G20570***	Plastocyanin-like	21.7	Y
83	M4CUC7	Bra007821	**AT2G25060**	Plastocyanin-like	19	Y
84	M4CUX4	Bra008019	**AT1G72230**	Plastocyanin-like	17.9	Y
99	M4D017	Bra009815	***AT5G25090***	Plastocyanin-like	20.1	Y
100	M4D168	Bra010217	***AT4G31840***	Plastocyanin-like	18.7	Y
111	M4D499	Bra011303	***AT4G31840***	Plastocyanin-like	18	Y
208[R]	M4DVJ5	Bra020539	AT5G26330	Plastocyanin-like	19.8	Y
234[R]	M4E588	Bra023942	***AT3G20570***	Plastocyanin-like	20.7	Y
235[R]	M4E5C7	Bra023981	***AT4G31840***	Plastocyanin-like	18.3	Y
248	M4E901	Bra025257	***AT3G27200***	Plastocyanin-like	19	Y
255[R]	M4EC32	Bra026341	**AT4G27520**	Plastocyanin-like	32.9	Y
266[R]	M4EG04	Bra027717	AT1G64640	Plastocyanin-like	19.8	Y
310	M4EW04	Bra032987	***AT3G27200***	Plastocyanin-like	17.6	Y
337	M4F623	Bra036532	***AT5G25090***	Plastocyanin-like	18.8	Y

(Continued) Table S1.

No.[a]	Access Number[b]	Gene Name	Homologous *A.thaliana*[c]	Description[d]	Mass (kDa)	SP[e]
18	M4C8C6	Bra000454	***AT2G47470***	Thioredoxin	39.5	Y
51[D]	M4CJS1	Bra004455	***AT2G47470***	Thioredoxin	39.4	Y
155	M4DGP0	Bra015665	AT1G77510	Thioredoxin	55.8	Y
249	M4E9X7	Bra025584	AT5G40370	Thioredoxin	11.8	N/S
**Proteases**						
97[D]	M4CYL3	Bra009310	AT5G07830	Peptidase	52.7	Y
72	M4CS78	Bra007070	***AT3G54400***	Peptidase A1	46.1	Y
91	M4CXQ9	Bra009006	***AT5G10770***	Peptidase A1	109.8	Y
149	M4DE98	Bra014819	***AT3G54400***	Peptidase A1	45.9	Y
220	M4E0M8	Bra022325	***AT3G18490***	Peptidase A1	51.9	Y
275	M4EIF3	Bra028568	***AT5G10770***	Peptidase A1	50	Y
346	M4F917	Bra037580	***AT3G18490***	Peptidase A1	52.6	Y
82	M4CU81	Bra007775	AT2G25940	Peptidase C13, legumain	53.3	Y
109	M4D419	Bra011223	***AT4G30810***	Peptidase S10	53.5	Y
196	M4DSC2	Bra019415	**AT3G45010**	Peptidase S10	56.8	Y
214	M4DXI4	Bra021230	*AT3G17180*	Peptidase S10	53.9	Y
309	M4EV48	Bra032681	**AT4G12910**	Peptidase S10	56.6	Y
320	M4EZY4	Bra034377	**AT2G27920**	Peptidase S10	51.5	Y
372	M4FIZ3	Bra041072	***AT1G11080***	Peptidase S10	53.9	Y
25[D]	M4CAY0	Bra001359	AT3G10410	Peptidase S10, serine carboxypeptidase	56.9	Y
245	M4E6L4	Bra024418	AT5G65760	Peptidase S28	95.3	Y
387	Q8W179	CP4	AT4G39090	Senescence-associated cysteine protease	40.2	Y
**Lipid metabolism**						
40	M4CFC2	Bra002904	AT5G55480	Glycerophosphoryl diester phosphodiesterase	82.9	Y
256	M4ECA0	Bra026409	**AT4G26690**	Glycerophosphoryl diester phosphodiesterase	82.4	Y/M
359[D]	M4FD02	Bra038972	AT1G54790	Lipase	41.8	Y
289	M4EN89	Bra030259	AT2G22170	Lipoxygenase	20.2	Y
103	M4D2G5	Bra010665	AT4G39730	Lipoxygenase, LH2	19.7	Y
67[D]	M4CR80	Bra006721	***AT5G59320***	Non-specific lipid-transfer protein	11.6	Y
205	M4DUX9	Bra020322	**AT5G59310**	Non-specific lipid-transfer protein	11.4	Y
206[D]	M4DUY0	Bra020323	***AT5G59320***	Non-specific lipid-transfer protein	11.3	Y
383	Q6IWH2	Bra017113	**AT2G38530**	Non-specific lipid-transfer protein	11.9	Y
2[D]	Q42641	WAX9A	**AT2G38540**	Non-specific lipid-transfer protein	11.9	Y
123	M4D6M6	Bra012135	AT5G67130	Phospholipase	46.7	Y
144[D]	M4DCJ4	Bra014209	AT1G49740	Phospholipase	39.6	Y
56	M4CKT8	Bra004824	**AT2G44300**	Plant lipid transfer protein	21.4	Y
131	M4D9H5	Bra013135	AT2G13820	Plant lipid transfer protein	17.3	Y
142[R]	M4DCE0	Bra014154	AT1G48750	Plant lipid transfer protein	10	Y
150	M4DED2	Bra014853	**AT3G53980**	Plant lipid transfer protein	13.5	Y
221	M4E0R7	Bra022364	AT3G18840	Plant lipid transfer protein	10.7	Y
322	M4F0H5	Bra034570	AT4G33550	Plant lipid transfer protein	12.2	Y

(Continued) Table S1.

No.[a]	Access Number[b]	Gene Name	Homologous *A.thaliana*[c]	Description[d]	Mass (kDa)	SP[e]
**Signaling**						
39	M4CF96	Bra002878	**AT5G55730**	FAS1 domain	44.8	Y
58	M4CL29	Bra004915	**AT2G45470**	FAS1 domain	42.7	Y
61	M4CNF2	Bra005740	**AT5G03170**	FAS1 domain	25.2	Y
79	M4CTM5	Bra007569	**AT3G60900**	FAS1 domain	43.6	Y
132	M4D9N7	Bra013197	**AT2G04780**	FAS1 domain	27.1	Y
153	M4DFK1	Bra015274	**AT1G03870**	FAS1 domain	26.4	Y
198	M4DSS9	Bra019572	***AT4G12730***	FAS1 domain	43	Y
262	M4EFG9	Bra027531	AT5G44130	FAS1 domain	26.4	Y
284	M4EMA5	Bra029925	***AT4G12730***	FAS1 domain	43	Y
**Interactig domains**						
48[R]	M4CHF2	Bra003635	***AT1G78850***	Curculin-like (mannose-binding) lectin	49	Y
325	M4F1X0	Bra035067	***AT1G78830***	Curculin-like (mannose-binding) lectin	50.3	Y
326	M4F1X3	Bra035070	***AT1G78850***	Curculin-like (mannose-binding) lectin	49.3	Y
260	M4EEP5	Bra027257	*AT3G16530*	Legume lectin	30.6	Y
192	M4DR69	Bra019012	AT1G53070	Legume lectin, beta chain	30.5	Y
299	M4ESL4	Bra031794	AT1G53080	Legume lectin, beta chain	31.9	Y
122[R]	M4D6D6	Bra012045	AT2G26730	Leucine-rich repeat	68.2	Y
268[D]	M4EGU1	Bra028006	AT1G33590	Leucine-rich repeat	52.1	Y
98[R]	M4CZL3	Bra009660	AT2G17120	Peptidoglycan-binding lysin domain	38.1	Y
369	M4FHS3	Bra040651	AT4G16500	Proteinase inhibitor I25	12.2	Y
117	M4D551	Bra011608	***AT4G35350***	Proteinase inhibitor I29	39.6	Y
141	M4DC45	Bra014057	**AT1G47128**	Proteinase inhibitor I29	50.6	Y
317[R]	M4EXW8	Bra033657	**AT5G43060**	Proteinase inhibitor I29	50.5	Y
388[D]	M4D6Z2	Bra012252	AT1G20850	Proteinase inhibitor I29	39.4	Y
170	M4DJA2	Bra016580	**AT1G17860**	Proteinase inhibitor I3	21.9	Y
124	M4D6P8	Bra012157	***AT5G67360***	Proteinase inhibitor I9	79.7	Y
152[D]	M4DFA0	Bra015172	AT2G05920	Proteinase inhibitor I9	81.1	Y
253	M4EAJ0	Bra025798	***AT1G20160***	Proteinase inhibitor I9	80.1	Y/-
311[R]	M4EWC9	Bra033113	***AT5G67360***	Proteinase inhibitor I9	72.9	Y
361	M4FD81	Bra039051	***AT5G67360***	Proteinase inhibitor I9	78.4	Y
129	M4D7J8	Bra012458	**AT1G26450**	X8	19.9	Y
**Miscellaneous**						
238	M4E5U6	Bra024150	AT4G29260	Acid phosphatase	28.8	Y
229	M4E300	Bra023151	AT1G30900	EGF-like calcium-binding	69.5	Y
8	C1KH67	ESM	AT3G14210	Epithiospecifier modifier	43.1	Y
313[D]	M4EWX9	Bra033314	AT1G02335	Germin	23.2	Y
81	M4CTV8	Bra007652	**AT3G62020**	Germin, manganese binding site	23.4	Y
332	M4F4J7	Bra035997	AT5G02500	Heat shock protein 70	16	Y
209	M4DVJ7	Bra020541	***AT5G26280***	MATH	39	Y
338	M4F664	Bra036573	***AT5G26280***	MATH	39.5	Y

(Continued) Table S1.

No.[a]	Access Number[b]	Gene Name	Homologous *A.thaliana*[c]	Description[d]	Mass (kDa)	SP[e]
358	M4FCX6	Bra038946	**AT5G34850**	Metallophosphoesterase	55.4	Y
368	M4FGY5	Bra040363	**AT3G07130**	Metallophosphoesterase	60.3	Y
4[D]	A6XG32		AT5G26000	Myrosinase	62.7	Y
5	B1N8M3		AT2G14580	Pathogenesis-related protein	17.8	Y
360[R]	M4FD61	Bra039031	AT2G19970	Pathogenesis-related protein 1	19.6	Y
265	M4EFW8	Bra027681	*AT1G64160*	Plant disease resistance response protein	20.7	Y
295	M4EQT8	Bra031161	AT2G21100	Plant disease resistance response protein	20.2	Y
385	Q8H6J9	Bra001122	AT3G04720	PR4-type protein	15.5	Y
116	M4D536	Bra011593	AT4G35220	Putative cyclase	30.2	Y
178	M4DMA1	Bra017633	***AT4G34180***	Putative cyclase	28.5	Y
128	M4D7H9	Bra012439	**AT1G26820**	Ribonuclease T2	25.4	Y
258	M4ECR0	Bra026570	**AT2G02990**	Ribonuclease T2	25.2	Y
49[R]	M4CJB0	Bra004294	AT1G68290	S1\/P1 nuclease	33	Y
172[D]	M4DK21	Bra016849	AT1G11190	S1\/P1 nuclease	30.8	Y
157	M4DH37	Bra015814	***AT1G75800***	Thaumatin	34.3	Y
85[D]	M4CVH7	Bra008224	***AT1G75800***	Thaumatin, pathogenesis-related	33.8	Y
159	M4DH97	Bra015874	AT1G75030	Thaumatin, pathogenesis-related	25.7	Y
312[R]	M4EWF4	Bra033138	**AT4G11650**	Thaumatin, pathogenesis-related	26.4	Y
**Unknown function**						
230	M4E3N1	Bra023384	**AT5G12950**	Protein of unknown function DUF1680	96.2	Y
297	M4ER98	Bra031321	**AT3G22060**	Protein of unknown function DUF26	27.8	Y
345	M4F8R8	Bra037480	**AT5G48540**	Protein of unknown function DUF26	28.7	Y
71	M4CRY9	Bra006981	AT3G53235	Uncharacterized protein	8.9	Y
108	M4D3Z1	Bra011195	*AT4G30450*	Uncharacterized protein	9.6	Y
211[R]	M4DW90	Bra020784	**AT3G06035**	Uncharacterized protein	21.9	Y
254	M4EAR4	Bra025873	AT1G21090	Uncharacterized protein	26.1	Y
290	M4EP06	Bra030526	**AT1G03220**	Uncharacterized protein	43.7	Y
**Intracellular**						
377	Q2A960	40.t00060	AT4G17830	Acetylornithine deacetylase	48	N
36	M4CEV5	Bra002737	***AT5G57330***	Aldose 1-epimerase	35.5	N
68[D]	M4CRI4	Bra006825	***AT5G57330***	Aldose 1-epimerase	35.4	N
168	M4DIN0	Bra016357	***AT1G23190***	Alpha-D-phosphohexomutase	63.4	N
247	M4E713	Bra024568	***AT1G23190***	Alpha-D-phosphohexomutase	63.4	N
324[R]	M4F144	Bra034790	***AT3G10740***	Alpha-L-arabinofuranosidase	19.2	N
139	M4DBU6	Bra013956	***AT4G26140***	Beta-galactosidase	81.7	N/M
163	M4DI58	Bra016185	AT1G70810	C2 membrane targeting protein	18.7	N/M
267	M4EGF3	Bra027867	AT1G58370	Carbohydrate-binding	102.5	N
55[D]	M4CKN8	Bra004773	***AT2G43620***	Chitin-binding	25.5	N
232[D]	M4E4E2	Bra023645	**AT5G17920**	Cobalamin (vitamin B12)-independent methionine synthase	84.4	N
187	M4DQ39	Bra018630	AT1G08370	Dcp1-like decapping	40.2	N

(Continued) Table S1.

No.[a]	Access Number[b]	Gene Name	Homologous *A.thaliana*[c]	Description[d]	Mass (kDa)	SP[e]
376[D]	Q14U56	ebm	**AT1G09010**	Endo-beta-mannosidase	107.6	N
224	M4E2S1	Bra023072	AT2G36530	Enolase	47.5	N
382	Q4TU02		**AT1G54040**	Epithiospecifier protein	37.7	N
370[D]	M4FHW6	Bra040695	AT4G15093	Extradiol ring-cleavage dioxygenase	30.1	N
69	M4CRW2	Bra006954	***AT3G52880***	FAD-dependent pyridine nucleotide-disulphide oxidoreductase	46.4	N
264[D]	M4EFV7	Bra027670	AT1G63940	FAD-dependent pyridine nucleotide-disulphide oxidoreductase	52.3	N/C
134	M4DAG6	Bra013476	***AT4G20830***	FAD-binding	59.6	N/C
70	M4CRW8	Bra006960	***AT3G52930***	Fructose-bisphosphate aldolase	40.5	N
274	M4EIC8	Bra028543	***AT3G52930***	Fructose-bisphosphate aldolase	38.4	N
145	M4DCX3	Bra014340	**AT2G01140**	Fructose-bisphosphate aldolase	42.3	N/C
252[R]	M4EAE1	Bra025749	AT1G19570	Glutathione S-transferase	23.5	N
379	Q39367		AT3G17050	Glycine-rich protein (Fragment)	15.4	N
215	M4DXP5	Bra021291	**AT3G18080**	Glycoside hydrolase, family 1	64.1	N
77	M4CSX2	Bra007314	***AT3G57260***	Glycoside hydrolase, family 17	24.3	N
101	M4D1I0	Bra010330	***AT4G29360***	Glycoside hydrolase, family 17	56	N
147	M4DDR8	Bra014636	***AT3G57260***	Glycoside hydrolase, family 17	34.3	N
314[D]	M4EWY3	Bra033318	AT1G02360	Glycoside hydrolase, family 19	21.5	N
306[R]	M4EU69	Bra032351	AT1G30000	Glycoside hydrolase, family 47	68.3	N
200	M4DTG8	Bra019811	AT1G13130	Glycoside hydrolase, family 5	58.3	N
29	M4CC86	Bra001816	***AT3G21160***	Glycoside hydrolase	64.8	N/M
223[R]	M4E1T6	Bra022736	AT5G54660	Heat shock protein Hsp20	20.7	N
11[D]	M1GDJ6	MDH	AT1G04410	Malate dehydrogenase	35.7	N
292	M4EQ80	Bra030951	***AT1G53240***	Malate dehydrogenase	35.7	N/M
362	M4FEZ0	Bra039662	***AT1G53240***	Malate dehydrogenase	35.7	N/M
190	M4DQZ7	Bra018940	**AT1G52030**	Mannose-binding lectin	124	N
246	M4E6W1	Bra024516	AT2G17420	Pyridine nucleotide-disulphide oxidoreductase	31.6	N/M
384[D]	Q75UU6	BO-MDAR	***AT3G52880***	Monodehydroascorbate reductase	46.5	N
286[D]	M4EMP9	Bra030069	AT1G27970	Nuclear transport factor 2	13.6	N
263[D]	M4EFL7	Bra027579	AT4G09320	Nucleoside diphosphate kinase	16.4	N
199[R]	M4DT26	Bra019669	AT1G14250	Nucleoside phosphatase GDA1\/CD39	52	N
47[R]	M4CHB4	Bra003597	AT1G79690	NUDIX hydrolase domain	85.4	N
277	M4EIK4	Bra028619	***AT5G09760***	Pectinesterase inhibitor	61.1	N
113	M4D4K8	Bra011412	**AT4G33090**	Peptidase M1	97.5	N
110	M4D420	Bra011224	***AT4G30810***	Peptidase S10	61	N
201	M4DTP0	Bra019883	***AT1G11080***	Peptidase S10	52.6	N
104[R]	M4D2M5	Bra010728	AT4G38740	Peptidyl-prolyl cis-trans isomerase	18.3	N
231[D]	M4E3U6	Bra023449	AT5G14130	Peroxidase	39.7	N
351	M4FBR0	Bra038526	***AT2G22420***	Peroxidase	34.9	N
114	M4D4N4	Bra011438	***AT4G33420***	Peroxidase	33.4	N/M

(Continued) Table S1.

No.[a]	Access Number[b]	Gene Name	Homologous *A.thaliana*[c]	Description[d]	Mass (kDa)	SP[e]
30[D]	M4CD35	Bra002116	***AT3G20570***	Plastocyanin-like	84.4	N
373	M4FJ39	Bra041119	AT2G15780	Plastocyanin-like	20.1	N/C
184[D]	M4DP08	Bra018248	AT2G31390	Probable fructokinase-1	35.1	N
303	M4ETC5	Bra032055	AT2G24940	Probable steroid-binding protein	11	N
38	M4CF22	Bra002804	AT5G56600	Profilin	14.1	N
105	M4D3P5	Bra011099	***AT2G19760***	Profilin	14.1	N
294	M4EQM6	Bra031097	***AT2G19760***	Profilin	14	N
16	M4C820	Bra000348	AT2G44230	Protein of unknown function	85.3	N
347	M4F997	Bra037660	AT2G44260	Protein of unknown function DUF946, plant	60.4	N
386[R]	Q8LK82	CND1	***AT5G10770***	Putative chloroplast nucleoid DNA-binding protein	17.2	N
137	M4DBE0	Bra013800	AT4G24430	Rhamnogalacturonate lyase	73.8	N
202	M4DTY5	Bra019978	AT1G09890	Rhamnogalacturonate lyase	72.5	N
140[D]	M4DBY9	Bra014001	AT4G13930	Serine hydroxymethyltransferase	47.5	N
32[D]	M4CDQ1	Bra002332	***AT5G20830***	Sucrose synthase	90.1	N/M
66[D]	M4CQT7	Bra006578	***AT5G20830***	Sucrose synthase	92.4	N/M
321	M4F001	Bra034394	AT2G28190	Superoxide dismutase	21.4	N/C
138[D]	M4DBK3	Bra013863	AT4G25100	Superoxide dismutase	23.8	N
298	M4ES64	Bra031642	AT1G08830	Superoxide dismutase	15.2	N
339	M4F6X9	Bra036839	AT3G51030	Thioredoxin	12.6	N
90	M4CX84	Bra008831	**AT5G13420**	Transaldolase	47.9	N/C
87	M4CW30	Bra008427	AT1G79620	Tyrosine-protein kinase	160.3	N
282	M4ELA2	Bra029570	AT4G05050	Ubiquitin	17.2	N
94	M4CY57	Bra009154	**AT5G05960**	Uncharacterized protein	7.6	N/C
43	M4CFR5	Bra003047	AT5G53550	Uncharacterized protein	88.8	N

a) Number refers to Supporting Information Tables S2. Number with [D] or [R] indicates identification only in Delicious or YCR-Rinen, respectively.

b) Access number represent an ID in Uniport website (http://www.uniprot.org/)

c) Genes given in bold and italic indicates a detection in Ligat et al (2011) and Kehr et al (2004), respectively

d) Description is either generated from Uniport (http://www.uniprot.org/) or Brassica database (http://brassicadb.org/brad/index.php) of InterPro domain annotation

e) The signal peptide (SP) was predicted using SignalP (http://www.cbs.dtu.dk/services/SignalP/) and TargetP (http://www.cbs.dtu.dk/services/TargetP/). When both predictions are consistent, only the SignalP result is shown. “Y” and “N” indicate whether or not the sequence contains a signal peptide in secretory pathway. When the predictions differ, both predictions are shown. “C” indicates that TargetP predicted a chloroplast transit peptide. “M” indicates that TargetP predicted a mitochondrial targeting peptide. “S” indicates that TargetP predicted a secretory pathway, the target contains a signal peptide. “-” indicates that TargetP predicted “any other location”.

Table S2. Foc infection causes lectin and LRR changes in *B. oleracea* xylem sap

No.[a]	Access Number[b]	Gene Name	*Homologous in A.thaliana*	Mass (kDa)	Describtion	Max fold change Del/Ri[c]
262	M4EEP5	Bra027257	AT3G16530	30.6	Legume lectin	-3.5/- 4.3
301	M4ESL4	Bra031794	AT1G53080	31.9	Legume lectin	-5.4/ 2.5
328	M4F1X3	Bra035070	AT1G78850	49.3	Curculin-like lectin	3.3/ –
215	M4DX55	Bra021101	AT3G16530	30.2	Legume lectin	2.7/ –
194	M4DR69	Bra019012	AT1G53070	30.5	Legume lectin	2.7/ 3.3
59	M4CME0	Bra005378	AT2G34930	99.8	Leucine-rich repeat	8.9/ 1.7
235	M4E571	Bra023925	AT3G20820	41.3	Leucine-rich repeat	2.1/ –
354	M4FC84	Bra038700	AT3G12145	36	Leucine-rich repeat	4.8/ –
270	M4EGU1	Bra028006	AT1G33590	52.1	Leucine-rich repeat	2.0/ 6.7
123	M4D6D6	Bra012045	AT2G26730	68.2	Leucine-rich repeat	–/- 3.78
167	M4DI99	Bra016226	AT1G56130	60.2	Leucine-rich repeat	–/ 6.7

a) Number refers to Supporting Information Tables S2.

b) Access number represent an ID in Uniport website (http://www.uniprot.org/)

c) Number with no mark indicates the inducible fold change, while negative number indicates suppressed in Delicious or Rinen. Hyphen indicates the protein or the protein fold change has not been detected.

Table S3. Foc infection causes thaumatin induction in *B. oleracea* xylem sap.

No.[a]	Access Number [b]	Gene Name	Homologous in *A.thaliana*	Mass (kDa)	Max fold change Del/Ri [c]
159	M4DH37	Bra015814	AT1G75800	34.3	-2.01/–
122	M4DH97	Bra015874	AT1G75030	25.6	2.6/ 6.9
106	M4DB14	Bra013674	AT4G11650	25.0	21.8/ 22.9
187	M4EAC0	Bra025728	AT1G19320	25.4	3.3/ 3.7
186	M4EA13	Bra025620	AT5G40020	28.2	–/ 5.6
233	M4EWF4	Bra033138	AT4G11650	26.4	7.3/ 3.2
64	M4CVH7	Bra008224	AT1G75800	33.8	2.0/ –

a) Number refers to Supporting Information Tables S2.

b) Access number represent an ID in Uniport website (http://www.uniprot.org/)

c) Number with no mark indicates the inducible fold change, while negative number indicates suppressed in Delicious or Rinen. Hyphen indicates the protein or the protein fold change has not been detected.

Table S4. Foc protein annotation based on the result of blast in NCBI database

Accession [a]	Description[b]	Max score	Total score	Query cover	E value	Identity	Accession
P00041/contig_75_2	hypothetical protein FOPG_18421	1158	1158	100%	0	99%	EXL65345.1
	Carboxylesterase, type B	581	581	97%	0	53%	EQL02703.1
P01134/contig_644_31	hypothetical protein FOXB_12272	1930	1930	100%	0	100%	EGU77195.1
	ferric reductase Fre2p	1385	1385	78%	0	91%	KLO82874.1
P01592/contig_663_2	hypothetical protein FOXB_11849	567	567	100%	0	100%	EGU77619.1
	Putative glycosidase crf1	565	565	100%	0	99%	EMT60935.1
P03403/contig_739_59	hypothetical protein FOXB_06651	284	284	100%	1.00E-95	100%	EGU82848.1
	**Protein SnodProt1**	278	278	100%	3.00E-93	99%	EMT63329.1
P04292/contig_761_13	hypothetical protein FOXB_10022	608	608	100%	0	100%	EGU79437.1
	endoglucanase c	605	605	100%	0	99%	KLO84549.1
P05132/contig_785_69	hypothetical protein FOPG_06838	602	602	100%	0	100%	EXL79069.1
	Valacyclovir hydrolase	599	599	100%	0	99%	EMT70625.1
P08654/contig_907_28	hypothetical protein FOXB_06362	1244	1244	100%	0	100%	EGU83101.1
	Alcohol dehydrogenase [acceptor]	1226	1226	98%	0	100%	ENH67326.1
P10310/contig_989_8	hypothetical protein FOXB_08454	798	798	100%	0	100%	EGU81045.1
	Putative serine-rich protein C13G6.10c	796	796	100%	0	99%	ENH61243.1
P10456/contig_995_21	hypothetical protein FOXB_02119	672	672	100%	0	100%	EGU87360.1
	**Endo-1,4-beta-xylanase C**	671	671	100%	0	99%	EMT71906.1
P11164/contig_1045_2	hypothetical protein FOXB_03520	905	905	100%	0	100%	EGU85964.1
	chitinase	904	904	100%	0	99%	EWZ81209.1
P11220/contig_1051_3	hypothetical protein FOXB_13680	771	771	100%	0	100%	EGU75813.1
	BNR/Asp-box repeat domain protein	761	761	100%	0	99%	KLO92858.1
P11242/contig_1054_1	hypothetical protein FOXB_07409*	236	236	100%	2.00E-77	100%	EGU82078.1
P11311/contig_1060_12	hypothetical protein FOXB_13122	539	539	100%	0	100%	EGU76372.1
	**LysM domain-containing protein**	195	340	99%	4.00E-56	40%	XP_007286381.1

(Continued) Table S4. Foc protein annotation based on the result of blast in NCBI database

Protein Name	Description	Max score	Total score	Query cover	E value	Ident	Accession
P11347/contig_1063_1	hypothetical protein FOXB_07875	1245	1245	100%	0	100%	EGU81608.1
	Secreted oxidoreductase ORX1-like protein	1231	1231	100%	0	99%	AKC01502.1
P13298/contig_1456_1	hypothetical protein FOXB_15742*	354	354	100%	2.00E-122	100%	EGU73748.1
P13299/contig_1456_2	hypothetical protein FOXB_15741*	469	469	100%	1.00E-165	96%	EGU73747.1
P13310/contig_1464_1	hypothetical protein FOXB_06593	699	699	98%	0	100%	EGU82892.1
	hatching enzyme	483	483	98%	1.00E-166	66%	ENH87624.1
P13373/contig_1509_1	hypothetical protein FOPG_19255*	549	549	100%	0	100%	EXL64481.1
P14728/contig_2465_1	**Fo5176-SIX4**	493	493	93%	1.00E-174	100%	EGU85276.1
P14743/contig_2555_1	hypothetical protein FOXB_06595	849	849	100%	0	100%	EGU82890.1
	hatching enzyme	621	621	100%	0	70%	ENH87624.1
P15123/contig_3892_1	hypothetical protein FOPG_17523	1031	1031	100%	0	100%	EXL66292.1
	FAD binding domain-containing protein	482	482	98%	1.00E-162	49%	XP_007590639.1
P15632/contig_3990_11	hypothetical protein FOXB_08234	861	861	100%	0	100%	EGU81246.1
	Chitinase A1	848	848	100%	0	99%	ENH75524.1
P15981/contig_4126_1	**Fo5176-SIX1**	586	586	100%	0	100%	EGU87748.1
P16246/contig_4338_1	hypothetical protein FOXB_06305	469	469	100%	8.00E-166	100%	EGU83183.1
	**Endo-1,4-beta-xylanase**	441	441	100%	1.00E-154	93%	EWG49249.1
P16923/contig_5980_1	hypothetical protein FOMG_19011*	315	315	100%	3.00E-107	91%	EXK24251.1

[a] Serial number/contig number of Foc Cong: 1-1 genome database used in this study. [b]Top one of the BLAST result for each protein was listed in the table. For those hypothetical proteins, a functional/ putative functional protein in the BLAST result with max score >150 and query cover >60% was also selected and listed as reference. Descriptions given in bold were discussed in the paper. Descriptions with an asterisk indicate BLAST result of the corresponding protein contains no reliable functional/ putative functional protein item. FOPG refers to *F. oxysporum* f. sp. *conglutinans* race 2 54008. FOXB refers to *F. oxysporum* Fo5176. FOMG refers to *F. oxysporum* f. sp. *melonis*.

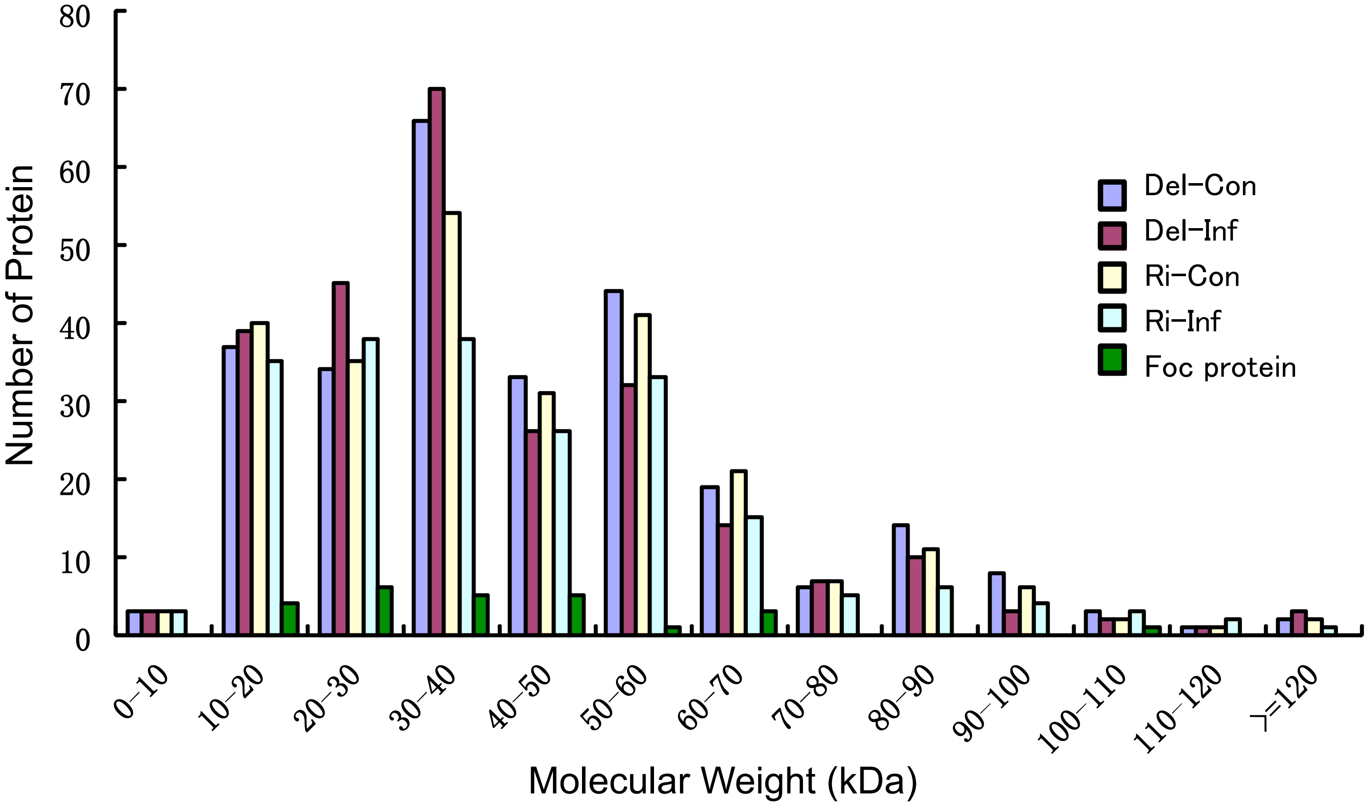

Figure S1. Distribution of molecular weight for proteins identified by the in-solution LC-MS/MS analysis for Foc-infected and non- infected *B. oleracea*.

1 **MVRLNQILLLAGNLLPVFG**AATKSSNSAKVPEPQQPGIVSNCKSYYLVEK 50

51 GETCSEVAAKNKISLSDFLEWNPKTGTDCNALLANAYACVSVTETKGSSS 100

101 AKPPAKKYSPTQAGIAKNCAKYALVGKTTTCKSIETQNKLSFANFYKWNP 150

151 AIGKHCQGLKKGYYVCVGVEKAATPTPTTPENVTGNKAPSPVQKGITKSC 200

201 NEYHRVSKGDTCSAVASEFNVDLAEFYEWNPAVGSKCENLWAGYYYCVRV 250

251 PGEKAKASVHTPSPN

Figure S2. Peptide sequence of Foc protein P11311. The sequence given in bold indicate signal peptide that predicted by SignalP (http://www.cbs.dtu.dk/services/SignalP/). Tryptic peptides identified in LC-MS/MS analysis are underlined. LysM domains are outlined by gray color.
